# Cost-effectiveness of neonatal surgery for congenital anomalies in low-income and middle-income countries: a systematic review protocol

**DOI:** 10.1136/bmjpo-2020-000755

**Published:** 2020-08-30

**Authors:** Na Eun Kim, Dominique Vervoot, Ahmad Hammouri, Cristiana Riboni, Hosni Salem, Caris Grimes, Naomi Jane Wright

**Affiliations:** 1 Department of General Surgery, Boston Medical Center, Boston, Massachusetts, USA; 2 King's College London, London, UK; 3 Department of Health Policy and Management, Johns Hopkins Bloomberg School of Public Health, Baltimore, Maryland, USA; 4 Department of Internal Medicine, Bethlehem Arab Society for Rehabilitation, Bethlehem, Palestine, State of; 5 University of Pavia, Pavia, Lombardia, Italy; 6 Cairo University, Giza, Egypt; 7 Department of Surgery, Medway NHS Foundation Trust, Gillingham, Kent, UK; 8 King’s Centre for Global Health and Health Partnerships, King’s College London, London, UK

**Keywords:** neonatology

## Abstract

**Introduction:**

Congenital anomalies are the fifth leading cause of death in children under 5 years old globally (591 000 deaths reported in 2016). Over 95% of deaths occur in low-income and middle-income countries (LMICs). It is estimated that two-thirds of the congenital anomaly health burden could be averted through surgical intervention and that such interventions can be cost-effective. This systematic review aims to evaluate current evidence regarding the cost-effectiveness of neonatal surgery for congenital anomalies in LMICs.

**Methods and analysis:**

A systematic literature review will be conducted in PubMed, MEDLINE, Embase, Cochrane Library, Scielo, Google Scholar, African Journals OnLine and Regional WHO’s African Index Medicus databases for articles on the cost-effectiveness of neonatal surgery for congenital anomalies in LMICs. The following search strings will be used: (1) congenital anomalies; (2) LMICs; and (3) cost-effectiveness of surgical interventions. Articles will be uploaded to Covidence software, duplicates removed and the remaining articles screened by two independent reviewers. Cost information for interventions or procedures will be extracted by country and condition. Outcome measurements by reported unit and cost-effectiveness ratios will be extracted. Methodological quality of each article will be assessed using the Drummond checklist for economic evaluations. The Agency for Healthcare Research and Quality’s Effective Health Care Program guidance will be followed to assess the grade of the studies.

**Ethics and dissemination:**

No ethical approval is required for conducting the systematic review. There will be no direct collection of data from individuals. The finalised article will be published in a scientific journal for dissemination. The protocol has been registered with PROSPERO (International Prospective Register of Systematic Reviews).

**Conclusion:**

Congenital anomalies form a large component of the global health burden that is amenable to surgical intervention. This study will systematically review the current literature on the cost-effectiveness of neonatal surgery for congenital anomalies in LMICs.

**PROSPERO registration number:**

CRD42020172971.

**What is already known on this topic?**

- Congenital anomalies are a large component of the global health burden, recently rising to become the fifth leading cause of death in children under 5 years globally.
- Over 95% of deaths occur in low-income and middle-income countries (LMICs).
- Surgical interventions have been shown to be cost-effective in reducing the burden of disease for some congenital anomalies.

**What this study hopes to add?**

- To provide an evidence-based summary of the cost-effectiveness of neonatal surgery for congenital anomalies in LMICs to direct future interventions and investments.
- To identify specific congenital conditions that are most cost-effective in LMICs.

## Introduction

Congenital anomalies constitute a large global health burden, accounting for an estimated 591 000 deaths worldwide in 2016.^1^ The burden of disease from congenital anomalies falls most heavily on low-income and middle-income countries (LMICs), where over 95% of the deaths from congenital anomalies occur.^2 3^ The burden of disease, traditionally expressed in disability-adjusted life years (DALYs), represents a health gap in a population due to early mortality and years of healthy life lost. As an example of this health gap, an estimated 140 154 DALYs are potentially avertable by neonatal surgery in Uganda; however, only 3.5% of the need is currently being met by the health system.^4^ A study of four low-income countries, Nepal, Rwanda, Sierra Leone and Uganda, showed 62% of the paediatric population had at least one unmet surgical need, approximating to 3.7 million children who need surgical care. These surgical conditions included congenital anomalies, along with masses, wounds, burns and abdominal pain. This study reported congenital anomalies were less likely to be treated compared with wounds, burns and extremity conditions.^5^ The higher burden in LMICs may also be attributed to a higher disease incidence related to a higher rate of micronutrient and macronutrient deficiencies, exposure to teratogens, intrauterine infections, and self-medication. In addition, elective pregnancy termination following prenatal diagnosis is less available in LMICs compared with high-income countries (HICs).^6^

Mortality rates for many congenital anomalies in LMICs are high. For example, gastroschisis mortality has been reported as 75%–100% in many paediatric surgical centres across Sub-Saharan Africa compared with less than 4% in HICs.^6–8^ Disturbingly, published high mortality rates from congenital anomalies may even be an underestimate of the true burden of disease due to sizeable hidden mortality. Children who do not reach health facilities and die at home or in transit are traditionally not accounted for.^9 10^ In 2014 the rate of facility-based delivery averaged 43% in developing countries and 48% in Sub-Saharan Africa.^11^ Barriers to facility-based delivery include social and cultural factors, distance to the facility, and cost of delivery.^12^ EUROCAT national estimates show that infant mortality from congenital anomalies could be up to 29% higher than that reported by the WHO.^13^ Furthermore, it is difficult to estimate the true burden of congenital anomalies in LMICs due to a lack of congenital anomaly registries and deficient research.

Traditionally, surgical care for neonates has been perceived as too complex and expensive to be prioritised or even implemented in LMICs, amidst competing public and global health priorities. However, a growing body of literature has arisen evaluating the potential of surgical interventions to reduce the burden of disease due to congenital anomalies in LMICs.^14–17^ Of DALYs caused by cleft lip and palate, congenital heart anomalies, and neural tube defects, 57% are potentially amenable to surgical care in LMICs.^14^ Among patients with gastroschisis in Uganda, up to 58.7 DALYs could be avertable per patient if appropriate surgical care was available.^18^ Despite this high unmet need that can be prevented with surgical care, the burden of surgical disease in the paediatric population remains high in LMICs. Many surgical conditions are congenital, yet surgical care in LMICs is often delayed, worsening outcomes due to presenting with more advanced disease processes.^15 19^ In Kenya, only 13.5% of the required surgical interventions were performed for common congenital conditions requiring surgical care.^17^

Surgical care has been found to be cost-effective in LMICs across various fields, including paediatric surgery.^20–22^ Treating congenital anomalies such as cleft lip and palate, anorectal malformation, congenital diaphragmatic hernia, and congenital heart defects can have significant economic impact by adding to lifetime individual income and quality life lived.^16 23–25^ In addition, untreated conditions such as hydrocephalus in infants can exact a large economic burden, whereas treating them has been shown to have a favourable cost:benefit ratio.^26^ Much has been added to the literature recently on the cost-effectiveness of surgery for paediatric conditions, including the systematic review by Saxton *et al* 22 in 2016 on the cost-effectiveness of surgery for paediatric conditions in LMICs. However, a systematic review of the cost-effectiveness studies for congenital anomalies in LMICs has not been conducted. Neonatal surgery is often deemed expensive due to the involvement of neonatal intensive care unit resources and specialists trained in neonates. Neonatal surgery uses a different set of resources and healthcare professionals compared with general paediatric surgery.

It is important to specifically analyse neonatal surgery independent of paediatric surgery and to fully understand the economic burden and the cost-effectiveness of surgical interventions for congenital anomalies to improve outcomes. This study aims to systematically review existing literature on the cost-effectiveness of neonatal surgery for the management of congenital anomalies in LMICs.

### Aim

The study aims to conduct a systematic review that identifies and analyses cost-effectiveness of neonatal surgical interventions for congenital anomalies in LMICs.

### Objectives

- To systematically identify studies on cost-effectiveness of neonatal surgery for congenital anomalies in LMICs.
- To evaluate the cost-effectiveness of neonatal surgery for congenital anomalies in LMICs.
- To provide an evidence-based summary of cost-effectiveness of neonatal surgery for congenital anomalies in LMICs to direct future interventions and investments in neonatal surgery.
- To identify specific congenital conditions that are most cost-effective in LMICs.
- To critically appraise the quality of the studies included in the systematic review.

## Methods and analysis

A systematic literature review will be conducted following the Preferred Reporting Items for Systematic Review and Meta-Analysis Protocols guidelines (online supplementary file 1). Any amendments to the protocol will be reported in the publication of the results.

10.1136/bmjpo-2020-000755.supp1 Supplementary data

### Inclusion/exclusion criteria

#### Inclusion criteria

- Conditions: abdominal wall defect, aganglionosis, anal atresia, anorectal malformation, anorectal stenosis, apple peel syndrome, biliary atresia, birth defects, bladder exstrophy, branchial tag, branchial vestige, bronchopulmonary sequestration, cervicoaural fistula, choledochal cyst, cleft lip, cleft palate, clubfoot, colonic atresia, congenital abnormalities, congenital anomalies, congenital cystic adenomatoid malformation of lung, congenital diaphragmatic hernia, congenital heart defects, congenital hydronephrosis, congenital malformation, conjoined twins, cryptorchidism, diaphragmatic eventration, Down syndrome, duodenal atresia, duodenal obstruction, duodenal web, epispadias, exomphalos, fetal malformation, gastroschisis, Hirschsprung’s disease, hydrocoele, hypospadias, ileal atresia, imperforate anus, imperforate hymen, indeterminate sex, intestinal atresia, jejunal atresia, jejuno-ileal atresia, malrotation, neural tube defects, oesophageal atresia, omphalocoele, orofacial clefts, pectus excavatum, pes cavus, phimosis, polycystic kidney disease, polydactyly, preauricular sinus, redundant neck fold, spina bifida, syndactyly, tongue tie, tracheo-oesophageal fistula, umbilical hernia, undescended testicle, volvulus and webbed neck.
- Age: neonatal.
- Place: LMICs (as defined by World Bank 2019 classification).
- Intervention: surgical or operative interventions performed in the antenatal setting and within the first 28 days of life.

#### Exclusion criteria

- Conditions: all conditions not listed in the inclusion criteria.
- Age: greater than 28 days of life.
- Place: HICs.
- Intervention: any procedure performed after the first 28 days of life.

### Search strategy

A systematic literature search will be conducted using the following search strings: (1) congenital anomalies; (2) LMICs; and (3) cost-effectiveness of surgical interventions (table 1). The following databases will be used: PubMed, MEDLINE, Embase, Cochrane Library, Scielo, Google Scholar, African Journals OnLine and Regional WHO’s African Index Medicus. The search will include English, French, Spanish, Italian and Arabic articles and will be restricted to human studies. There will be no restrictions on publication date or study design (online supplemental file 2).

10.1136/bmjpo-2020-000755.supp2 Supplementary data

**Table 1:** Search strategy

Search string 1	Search string 2	Search string 3
Congenital abnormalities, congenital anomalies, congenital malformation, abdominal wall defect, aganglionosis, anal atresia, anal stenosis, anorectal malformation, anorectal stenosis, apple peel syndrome, biliary atresia, birth defects, bladder exstrophy, branchial tag, branchial vestige, bronchopulmonary sequestration, bronchogenic cyst, cervicoaural fistula, choledochal cyst, cleft lip, cleft palate, clubfoot, colonic atresia, congenital cystic adenomatoid malformation of lung, congenital diaphragmatic hernia, congenital heart defects, congenital hydronephrosis, conjoined twins, cryptorchidism, diaphragmatic eventration, Down syndrome, duodenal atresia, duodenal obstruction, duodenal web, epispadias, exomphalos, fetal malformation, gastroschisis, Hirschsprung’s disease, hydrocele, hypospadias, ileal atresia, imperforate anus, imperforate hymen, indeterminate sex, intestinal atresia, jejunal atresia, jejuno-ileal atresia, malrotation, maxillofacial abnormalities, megacolon, mouth abnormalities, neural tube defects, oesophageal atresia, omphalocele, orofacial clefts, pectus excavatum, pes cavus, phimosis, polycystic kidney disease, polydactyly, preauricular sinus, rectosigmoid aganglionosis, redundant neck fold, renal anomalies, spina bifida, syndactyly, tongue tie, tracheo-oesophageal fistula, umbilical hernia, undescended testicle, urogenital abnormalities, volvulus, webbed neck.	LMICs, low- and middle-income countries, developing countries, low resource settings, underdeveloped countries, low-income countries, middle-income countries, limited resource settings, Africa South of the Sahara, Sub-Saharan Africa, less resourced communities, Afghanistan, Albania, Algeria, American Samoa, Angola, Argentina, Armenia, Azerbaijan, Bangladesh, Belarus, Belize, Benin, Bhutan, Bolivia, Bosnia and Herzegovina, Botswana, Brazil, Bulgaria, Burkina Faso, Burundi, Cabo Verde, Cambodia, Cameroon, Central African Republic, Chad, China, Colombia, Comoros, Democratic Republic of the Congo, DRC, Republic of the Congo, Costa Rica, Cote d’Ivoire, Ivory Coast, Cuba, Djibouti, Dominica, Dominican Republic, Ecuador, Egypt, Egypt Arab Republic, El Salvador, Equatorial Guinea, Eritrea, Eswatini, Ethiopia, Fiji, Gabon, Gambia, Georgia, Ghana, Grenada, Guatemala, Guinea, Guinea-Bissau, Guyana, Haiti, Honduras, India, Indonesia, Islamic Republic of Iran, Iraq, Jamaica, Jordan, Kazakhstan, Kenya, Kiribati, Democratic People’s Republic of Korea, Kosovo, Kyrgyz Republic, Lao PDR, Laos, Lebanon, Lesotho, Liberia, Libya, Madagascar, Malawi, Malaysia, Maldives, Mali, Marshall Islands, Mauritania, Mauritius, Mexico, Micronesia, Moldova, Mongolia, Montenegro, Morocco, Mozambique, Myanmar, Namibia, Nauru, Nepal, Nicaragua, Niger, Nigeria, North Macedonia, Pakistan, Papua New Guinea, Paraguay, Peru, Philippines, Romania, Russian Federation, Rwanda, Samoa, Sao Tome and Principe, Senegal, Serbia, Sierra Leone, Solomon Islands, Somalia, South Africa, South Sudan, Sri Lanka, Saint Lucia, Saint Vincent and the Grenadines, Sudan, Suriname, Swaziland, Syrian Arab Republic, Tajikistan, Tanzania, Thailand, Timor-Leste, Togo, Tonga, Tunisia, Turkey, Turkmenistan, Tuvalu, Uganda, Ukraine, Uzbekistan, Vanuatu, Venezuela, Vietnam, West Bank and Gaza, Republic of Yemen, Zambia, Zimbabwe, Kabul, Porto-Novo, Hogbonou, Adjace, Cotonou, Kutonu, Ouagadougou, Ouaga, Bujumbura, Usumbura, Phnom Penh, Bangui, Bangi, N’Djamena, Ndjamena, Fort Lamy, Moroni, Kinshasa, Asmara, Asmera, Addis Ababa, Addis Abeba, Banjul, Bathurst, Conakry, Bissau, Port-au-Prince, Pyongyang, Monrovia, Antananarivo, Tananarive, Tana, Lilongwe, Bamako, Maputo, Lourenco Marques, Kathmandu, Niamey, Kigali, Freetown, Free-town, Mogadishu, Xamar, Hamar, Muqdisho, Maqadishu, Juba, Dodoma, Dar es Salaam, Lome, Kampala, Harare, Salisbury, Yerevan, Dhaka, Dacca, Thimphu, Thimbu, Sucre, Charcas, La Plata, Chuquisaca, La Paz, Praia, Yaounde, Jaunde, Brazzaville, Yamoussoukro, Cairo, Accra, Tegucigalpa, Tegus, New Delhi, Jakarta, Nairobi, South Tarawa, Tarawa Teinainano, Pristina, Prishtina, Bishkek, Pishpek, Frunze, Vientiane, Maseru, Nouakchott, Palikir, Chisinau, Kishinev, Rabat, Nay Pyi Taw, Naypyidaw, Nepranytau, Naypyitaw, Kyetpyay, Pyinmana, Kyatpyay, Pyinmana, Yangon, Rangoon, Managua, Abuja, Lagos, Islamabad, Port Moresby, Moresby, Pom Town, Manila, Apia, Dakar, Honiara, Jayawardenepura, Jayewardenepura, Khartoum, Mbabane, Embabane, Lobamba, Damascus, Dushanbe, Dyushambe, Stalinabad, Dili, Kyiv, Kiev, Tashkent, Toshkent, Port Vila, Hanoi, Ha Noi, Sana’a, Sanaa, Sana, Lusaka, Ulaanbaatar, Ulan-Bator, Luanda, Tbilisi, Amman.	Cost, cost effectiveness, cost benefit analysis, cost of illness, cost effective surgical intervention, health care cost, DALYs, disability adjusted life years, QALYs, quality adjusted life years, HALYs, health adjusted life years.

The search strings were used during the literature search for this systematic review. Search string 1 encompasses congenital anomalies found in neonates that commonly require surgical intervention. Search string 2 includes all low-income and middle-income countries. Search string 3 describes the cost-effectiveness of an intervention.

### Study design

Published, peer-reviewed journal articles will be included. Any study without explicit cost data or health outcomes data will be excluded. Case reports, editorials, letters to the editor and literature reviews will be excluded. Abstracts without available full text will be excluded.

### Methodological quality

The studies will be assessed for their methodological quality of economic evaluations using the Drummond 10-point checklist^27^ (table 2). The results will be summarised in a table format in the results section. In addition to the Drummond checklist, the Agency for Healthcare Research and Quality’s Effective Health Care Program guidance will be followed to assess the grade of the studies. The domains included in this assessment are risk of bias, consistency, directness and precision. The strength of evidence will be categorised into high, moderate low and insufficient groups.^28^

**Table 2:** Drummond checklist

Item		Yes (page number)	No	Not clear	Not appropriate
Study design					
1	The research question is stated.				
2	The economic importance of the research question is stated.				
3	The viewpoint(s) of the analysis are clearly stated and justified.				
4	The rationale for choosing alternative programmes or interventions compared is stated.				
5	The alternatives being compared are clearly described.				
6	The form of economic evaluation used is stated.				
7	The choice of form of economic evaluation is justified in relation to the questions addressed.				
Data collection					
8	The source(s) of effectiveness estimates used are stated.				
9	Details of the design and results of effectiveness study are given (if based on a single study).				
10	Details of the methods of synthesis or meta-analysis of estimates are given (if based on a synthesis of a number of effectiveness studies).				
11	The primary outcome measure(s) for the economic evaluation are clearly stated.				
12	Methods to value benefits are stated.				
13	Details of the subjects from whom valuations were obtained were given.				
14	Productivity changes (if included) are reported separately.				
15	The relevance of productivity changes to the study question is discussed.				
16	Quantities of resource use are reported separately from their unit costs.				
17	Methods for the estimation of quantities and unit costs are described.				
18	Currency and price data are recorded.				
19	Details of currency of price adjustments for inflation or currency conversion are given.				
20	Details of any model used are given.				
21	The choice of model used and the key parameters on which it is based on justified.				
Analysis and interpretation of results					
22	Time horizon of costs and benefits is stated.				
23	The discount rate(s) is stated.				
24	The choice of discount rate(s) is justified.				
25	An explanation is given if costs and benefits are not discounted.				
26	Details of statistical tests and confidence intervals are given for stochastic data.				
27	The approach to sensitivity analysis is given.				
28	The choice of variables for sensitivity analysis is justified.				
29	The ranges over which the variables are varied are justified.				
30	Relevant alternatives are compared.				
31	Incremental analysis is reported.				
32	Major outcomes are presented in a disaggregated as well as aggregated form.				
33	The answer to the study question is given.				
34	Conclusions follow from the data reported.				
35	Conclusions are accompanied by the appropriate caveats.				

### Study screening

Articles will be uploaded to Covidence software (Melbourne, Australia), duplicate articles will be removed and the remaining articles will be screened by two independent reviewers. All potential eligible articles will be screened in full text for final selection by two independent reviewers. The reference lists of the included articles will be screened to identify further eligible studies. Conflicts between the two reviewers’ assessments will be resolved by a consensus meeting of all authors.

### Data extraction

The following data will be extracted: study type or design, study population, study period, country, year of publication, journal, author name(s), number of patients, patient demographics, type of condition, type of intervention, type of healthcare system, gestational age, weight, time from birth to presentation, costs incurred during treatment and outcome of intervention (mortality and morbidity which will be reported using the Clavien-Dindo scoring system).^29^

Main outcomes will be the cost of surgical interventions and health outcomes. The effectiveness method of DALYs, health-adjusted life years and quality-adjusted life years will be extracted. Reported incremental cost-effectiveness ratio and/or potential gains in life expectancy will be extracted. Studies without a comparison with a prior intervention and reporting only a cost-effectiveness ratio will also be included in our study. Data reported for different countries or procedures will be extracted as separate results.

The WHO-CHOICE guidelines will be used to determine the interventions’ cost-effectiveness category. Cost-effective intervention thresholds will be defined by the WHO based on the gross domestic product (GDP) per capita per DALY averted. Those that cost less than the GDP per capita per DALY averted will be categorised as very cost-effective, interventions that cost one to three times the GDP per capita per DALY as cost-effective, and those that cost more than three times the GDP per capita per DALY will be determined as not cost-effective.^30^

### Data synthesis

The data will be organised into author(s), year of publication, intervention or procedure, country or site of intervention, GDP, costs per outcome, outcome unit, and currency.

Cost data will be organised into cost to the provider and cost to the patient. The cost will be converted to US dollars, calculated by the currency year. Quantitative analysis will be undertaken by calculating the median values and IQR for the intervention or procedures. Meta-analysis will likely not be feasible due to the limited availability of data. If our search results in appropriate data, a meta-analysis will be conducted. Appropriate data is defined as two or more homogeneous studies comparing the cost and outcomes of a specific surgical procedure with another non-surgical intervention. The meta-analysis will be conducted in Stata 15.1.

The GRADE (Grading of Recommendations, Assessment, Development and Evaluations) system will be used to rate the quality of evidence and the strength of recommendations.

### Patient and public involvement

While we recognise the importance of public and patient involvement in research, on this occasion it has not been feasible to incorporate this into the design of the study due to difficulty engaging with patients and their families affected by congenital anomalies within LMICs in such a project. We will endeavour to ensure that a summary of the results of the study is provided in lay language and disseminated for public viewing.

### Ethics and dissemination

No ethical approval was required for conducting the systematic review. There was no direct collection of data from individuals. The finalised article will be published in a scientific journal for dissemination.

### Limitations

The studies may have been conducted in different settings, such as government facilities, non-profit hospitals and surgical mission trips, which may limit direct comparisons. Costs may also be reported that vary in the components included. In addition, the health outcome calculations will be different by study, which may or may not include age weighting or discounting. The search engines used may not identify all articles. Lastly, it is unlikely that the incremental cost-effectiveness ratio will be directly comparable among studies as the alternative comparison may be different for each study.

## Discussion

Congenital anomalies are reported to account for approximately 62.9 million or 2.4% of global DALYs in 2016.^31^ Published literature from LMICs commonly describes the burden of disease from congenital disease in terms of mortality instead of DALYs, emphasising the difference in mortality rates compared with those from HICs. Reducing infant mortality due to congenital anomalies will help to meet the Sustainable Development Goal to reduce preventable deaths in neonates to as low as 12 per 1000 live births by 2030.^32^ Current literature has shown that surgical care for congenital anomalies can reduce the burden of disease in a cost-effective manner.^16 21 23 24 33^ An estimated two-thirds of the burden of disease related to congenital anomalies can be averted through surgical care.^34^ Despite this potential, the burden of surgical disease in this neonatal population remains high in LMICs.

There is a growing body of literature on the cost-effectiveness of surgical interventions for congenital anomalies, but to our knowledge there has been no systematic analysis consolidating these studies. The most recent literature review by Stolk *et al* 33 was completed in 2000 reporting two complete economic evaluations of the cost-effectiveness of neonatal surgery for congenital anomalies which were not done in the context of LMICs.^35 36^ This project will provide a crucial addition to the literature by providing a systematic review on current literature of cost-effectiveness analysis of neonatal surgery for congenital anomalies in LMICs.

## Supplementary Material

Author's manuscript
