# Supplementary material for: Cost-effectiveness of neonatal surgery for congenital anomalies in low-income and middle-income countries: a systematic review protocol
Source: BMJ Paediatr Open. 2020 Aug 30;4(1):e000755. doi: 10.1136/bmjpo-2020-000755 (PMC7462241; doi:10.1136/bmjpo-2020-000755)
Supplement: Supplementary data [file bmjpo-2020-000755supp002.pdf]

Supplementary File 2b

Cost-Effectiveness of Neonatal Surgery for Congenital Anomalies in Low- and Middle-Income Countries: A Systematic Review Protocol

Database: Ovid MEDLINE(R) and Epub Ahead of Print, In-Process & Other Non-Indexed Citations, Daily and Versions(R) (from 1946 to March 2020)

Search strategy:

Search Number	Search term	Results
1	cost benefit analys*.mp.	83470
2	cost effectiveness.mp.	60533
3	cost of illness.mp.	28218
4	DALY*.mp.	3158
5	Disability Adjusted Life Year*.mp.	3402
6	HALY*.mp.	618
7	Health Adjusted Life Year*.mp.	49
8	Health Care Cost*.mp.	50825
9	QALY*.mp.	10711
10	Quality Adjusted Life Year*.mp.	18335
**11**	**1 or 2 or 3 or 4 or 5 or 6 or 7 or 8 or 9 or 10**	**179488**
12	exp Congenital Abnormalities/ or congenital anomal*.mp.	600611
13	exp Congenital Abnormalities/ or Congenital Abnormal*.mp.	595316
14	Congenital Malformation*.mp.	15162
15	Abdominal Wall Defect*.mp.	2217
16	Aganglionosis.mp.	1273
17	Anal Atresia.mp. or exp Anus, Imperforate/	2904
18	Imperforate Anus.mp. or exp Anus, Imperforate/	3103
19	Anorectal Malformation*.mp.	2208
20	Anorectal stenosis.mp.	71
21	Apple peel syndrome.mp.	18
22	Biliary atresia.mp. or exp Biliary Atresia/	4957
23	Birth Defect*.mp.	10388
24	Bladder Exstrophy.mp.	2590
25	Branchial Tag.mp.	0
26	Branchial Vestige.mp.	0
27	exp Bronchopulmonary Sequestration/ or Bronchopulmonary sequestration*.mp.	2122
28	exp Bronchogenic Cyst/ or Bronchogenic Cyst*.mp.	1924

29	Cervicoaural Fistula.mp.	3
30	exp Choledochal Cyst/ or Choledochal Cyst*.mp.	3001
31	Cleft Lip.mp.	18452
32	Cleft Palate.mp.	24399
33	Clubfoot.mp.	4358
34	Colonic Atresia.mp.	160
35	exp "Cystic Adenomatoid Malformation of Lung, Congenital"/ or Congenital Cystic adenomatoid malformation of lung*.mp.	948
36	exp Hernias, Diaphragmatic, Congenital/ or congenital diaphragmatic hernia*.mp.	6097
37	exp Heart Defects, Congenital/ or Congenital Heart Defect*.mp.	153690
38	Congenital Hydronephrosis.mp.	363
39	Conjoined Twins.mp. or exp Twins, Conjoined/	2111
40	Cryptorchidism.mp.	10253
41	exp Diaphragmatic Eventration/ or Diaphragmatic Eventration*.mp.	991
42	Down Syndrome.mp.	28528
43	Duodenal atresia.mp.	903
44	Duodenal Obstruction.mp.	3962
45	Duodenal Web.mp.	107
46	Epispadias.mp.	1149
47	Exomphalos.mp. or exp Hernia, Umbilical/	3920
48	Fetal Malformation*.mp.	2053
49	Gastroschisis.mp. or exp GASTROSCHISIS/	2516
50	Hirschsprung disease.mp. or exp Hirschsprung Disease/	4949
51	Hydrocele.mp.	3159
52	Hypospadias.mp.	7140
53	Ileal Atresia.mp.	271
54	Imperforate Anus.mp. or exp Anus, Imperforate/	3103
55	Imperforate Hymen.mp.	307
56	Intestinal Atresia.mp. or exp Intestinal Atresia/	2680
57	Indeterminate Sex.mp.	24
58	Jejunal Atresia.mp.	281
59	Jejuno-Ileal Atresia.mp.	44
60	Malrotation.mp.	2637
61	exp Maxillofacial Abnormalities/ or Maxillofacial Abnormalit*.mp.	29974
62	exp Megacolon/ or Ganglionic Megacolon.mp.	7382
63	exp Mouth Abnormalities/ or Mouth Abnormalit*.mp.	26084
64	exp Neural Tube Defects/ or Neural Tube Defect*.mp.	30773
65	Esophageal Atresia.mp. or exp Esophageal Atresia/ or ?esophageal Atresia.mp.	4500

66	Omphalocele.mp. or exp Hernia, Umbilical/	4485
67	Orofacial Cleft*.mp.	1444
68	Orofacial Cleft*.mp.	1444
69	Pes Cavus.mp.	624
70	Phimosis.mp.	1416
71	Polycystic Kidney Disease.mp.	7924
72	Polydactyly.mp.	3539
73	Preauricular Sinus.mp.	95
74	Rectosigmoid aganglionosis.mp.	19
75	Redundant Neck Fold.mp.	0
76	Renal Anomal*.mp.	1313
77	Spina Bifida.mp.	8781
78	syndactyly.mp	3643
79	Tongue Tie.mp.	269
80	exp Tracheoesophageal Fistula/ or Tracheo-?esophageal fistula*.mp.	3875
81	Umbilical Hernia.mp. or exp Hernia, Umbilical/	4483
82	Undescended Testicle.mp.	268
83	exp Urogenital Abnormalities/ or Genito-urinary Anomalies.mp.	61092
84	Volvulus.mp. or exp Intestinal Volvulus/	9614
85	Webbed Neck.mp.	210
86	Pectus Excavatum.mp.	2126
**87**	**12 or 13 or 14 or 15 or 16 or 17 or 18 or 19 or 20 or 21 or 22 or 23 or 24 or 25 or 26 or 27 or 28 or 29 or 30 or 31 or 32 or 33 or 34 or 35 or 36 or 37 or 38 or 39 or 40 or 41 or 42 or 43 or 44 or 45 or 46 or 47 or 48 or 49 or 50 or 51 or 52 or 53 or 54 or 55 or 56 or 57 or 58 or 59 or 60 or 61 or 62 or 63 or 64 or 65 or 66 or 67 or 68 or 70 or 71 or 72 or 73 or 74 or 75 or 76 or 77 or 78 or 79 or 80 or 81 or 82 or 83 or 84 or 85 or 96**	**674240**
88	LMICs.mp.	3732
89	(low- and middle-income countr*).mp.	17446
90	exp Developing Countries/ or Developing countr*.mp.	126283
91	Low-resource setting*.mp.	4109
92	Underdeveloped Countries.mp.	922
93	low-income countries.mp.	6145
94	middle-income countries.mp.	17641

95	limited resource setting*.mp.	391
96	Africa South of the Sahara.mp. or exp "Africa South of the Sahara"/	209030
97	Sub-Saharan Africa.mp. or exp "Africa South of the Sahara"/	217226
98	Less Resourced communities.mp.	2
99	Afghanistan.mp. or exp AFGHANISTAN/	6919
100	Albania.mp. or exp ALBANIA/	1357
101	Algeria.mp. or exp ALGERIA/	4233
102	American Samoa.mp.	397
103	Angola.mp. or exp ANGOLA/	1623
104	Argentina.mp. or exp ARGENTiNA/	22557
105	Armenia.mp. or exp ARMENIA/	1890
106	Azerbaijan.mp. or exp AZERBAIJAN/	1973
107	Bangladesh.mp. or exp BANGLADESH/	15564
108	Belarus.mp. or exp "Republic of Belarus"/	2704
109	Belize.mp. or exp BELIZE/	967
110	Benin.mp. or exp BENIN/	3906
111	exp BHUTAN/ or Bhutan.mp.	843
112	Bolivia.mp. or exp BOLIVIA/	4085
113	(Bosnia and Herzegovina).mp.	2812
114	Botswana.mp. or exp BOTSWANA/	2698
115	Brazil.mp. or exp BRAZIL/	121099
116	Bulgaria.mp. or exp BULGARIA/	8107

117	Burkina Faso.mp. or exp Burkina Faso/	4591
118	Burundi.mp. or exp BURUNDI/	980
119	Cabo Verde.mp. or exp Cabo Verde/	270
120	Cambodia.mp. or exp CAMBODIA/	4833
121	Cameroon.mp. or exp CAMEROON/	7932
122	Central African Republic.mp. or exp Central African Republic/	1216
123	Chad.mp. or exp CHAD/	1317
124	China.mp. or exp CHINA/	269542
125	Colombia.mp. or exp COLOMBIA/	15284
126	Comoros.mp. or exp COMOROS/	506
127	Democratic Republic of the Congo.mp. or exp "Democratic Republic of the Congo"/	5337
128	exp "Democratic Republic of the Congo"/ or DRC.mp.	5579
129	Republic of the Congo.mp. or exp Congo/	7191
130	Costa Rica.mp. or exp Costa Rica/	5719
131	Cote d'Ivoire.mp. or exp Cote d'Ivoire/	3946
132	Ivory Coast.mp. or exp Cote d'Ivoire/	3747
133	Cuba.mp. or exp CUBA/	6860
134	Djibouti.mp. or exp DJIBOUTI/	429
135	Dominica.mp. or exp DOMINICA/	501
136	Dominican Republic.mp. or exp Dominican Republic/	2442
137	Ecuador.mp. or exp ECUADOR/	5777

138	exp EGYPT/ or Egypt.mp.	21581
139	El Salvador.mp. or exp El Salvador/	1469
140	Equatorial Guinea.mp. or exp Equatorial Guinea/	481
141	Eritrea.mp. or exp ERITREA/	640
142	Eswatini.mp.	636
143	Ethiopia.mp. or exp ETHIOPIA/	18047
144	Fiji.mp. or exp FIJI/	1950
145	Gabon.mp. or exp GABON/	2127
146	exp GAMBIA/ or Gambia.mp.	3191
147	exp "GEORGIA (REPUBLIC)"/ or exp GEORGIA/ or Georgia.mp.	17363
148	Ghana.mp. or exp GHANA/	11902
149	Grenada.mp. or exp GRENADA/	349
150	Guyana.mp. or exp GUYANA/	1268
151	Haiti.mp. or exp HAITI/	4162
152	Honduras.mp. or exp HONDURAS/	2019
153	India.mp. or exp INDIA/	147631
154	Indonesia.mp. or exp INDONESIA/	16328
155	exp Iran/ or Islamic Republic of Iran.mp.	26832
156	exp IRAQ/ or Iraq.mp.	10229
157	Jamaica.mp. or exp JAMAICA/	4675
158	Jordan.mp. or exp JORDAN/	7350
159	Kazakhstan.mp. or exp KAZAKHSTAN/	3622

160	Kenya.mp. or exp KENYA/	21964
161	Kiribati.mp. or exp Micronesia/	2104
162	Democratic People's Republic of Korea.mp. or exp "Democratic People's Republic of Korea"/	300
163	Kosovo.mp. or exp KOSOVO/	962
164	Kyrgyz Republic.mp. or exp Kyrgyzstan/	1312
165	exp Laos/ or Lao PDR.mp.	2206
166	Laos.mp. or exp LAOS/	2903
167	Lebanon.mp. or exp LEBANON/	6114
168	Lesotho.mp. or exp LESOTHO/	762
169	Liberia.mp. or exp LIBERIA/	1920
170	Libya.mp. or exp LIBYA/	1719
171	Madagascar.mp. or exp MADAGASCAR/	5383
172	Malawi.mp. or exp MALAWI/	7731
173	Malaysia.mp. or exp MALAYSIA/	21648
174	Maldives.mp. or exp Indian Ocean Islands/	11983
175	Mali.mp. or exp MALI/	4050
176	Marshall Islands.mp. or exp Micronesia/	2112
177	Mauritania.mp. or exp MAURITANIA/	724
178	Mauritius.mp. or exp MAURITIUS/	1090
179	exp MEXICO/ or Mexico.mp.	60493
180	Micronesia.mp. or exp MICRONESIA/	2424

181	Moldova.mp. or exp MOLDOVA/	1021
182	Mongolia.mp. or exp MONGOLIA/	4670
183	Montenegro.mp. or exp MONTENEGRO/	870
184	Morocco.mp. or exp MOROCCO/	7918
185	Mozambique.mp. or exp MOZAMBIQUE/	3923
186	Myanmar.mp. or exp MYANMAR/	4187
187	Namibia.mp. or exp NAMIBIA/	1807
188	Nauru.mp. or exp Micronesia/	2064
189	Nepal.mp. or exp NEPAL/	11583
190	Nicaragua.mp. or exp NICARAGUA/	2202
191	exp NIGER/ or Niger.mp.	13783
192	Nigeria.mp. or exp NIGERIA/	37370
193	North Macedonia.mp.	599
194	Pakistan.mp. or exp PAKISTAN/	24741
195	Papua New Guinea.mp. or exp Papua New Guinea/	5354
196	Paraguay.mp. or exp PARAGUAY/	1676
197	Peru.mp. or exp PERU/	13308
198	Philippines.mp. or exp PHILIPPINES/	12053
199	Romania.mp. or exp ROMANIA/	12624
200	exp Russia/ or Russian Federation.mp.	54195
201	Rwanda.mp. or exp RWANDA/	3505
202	exp AMERICAN SAMOA/ or Samoa.mp. or exp SAMOA/ or exp	1151

	"INDEPENDENT STATE OF SAMOA"/	
203	(Sao Tome and Principe).mp.	163
204	Senegal.mp. or exp SENEGAL/	7737
205	Serbia.mp. or exp SERBIA/	5420
206	Sierra Leone.mp. or exp Sierra Leone/	2509
207	Solomon Islands.mp. or exp Melanesia/	6738
208	Somalia.mp. or exp SOMALIA/	2261
209	South Africa.mp. or exp South Africa/	53905
210	South Sudan.mp. or exp South Sudan/	546
211	Sri Lanka.mp. or exp Sri Lanka/	8505
212	Saint Lucia.mp. or exp Saint Lucia/	126
213	(Saint Vincent and the Grenadines).mp.	65
214	exp SUDAN/ or Sudan.mp.	9410
215	Suriname.mp. or exp SURINAME/	1216
216	Swaziland.mp. or exp SWAZILAND/	947
217	Syrian Arab Republic.mp.	85
218	Syria.mp. or exp SYRIA/	3095
219	Tajikistan.mp. or exp TAJIKISTAN/	1034
220	Tanzania.mp. or exp TANZANIA/	15184
221	Thailand.mp. or exp THAILAND/	36416
222	exp Timor-Leste/ or Timor-Leste.mp.	417
223	East Timor.mp.	208

224	Togo.mp. or exp TOGO/	1717
225	Tonga.mp. or exp TONGA/	505
226	Tunisia.mp. or exp TUNISIA/	10348
227	exp TURKEY/ or Turkey.mp.	53356
228	Turkmenistan.mp. or exp TURKMENISTAN/	777
229	Tuvalu.mp. or exp Micronesia/	2035
230	Uganda.mp. or exp UGANDA/	16683
231	Ukraine.mp. or exp UKRAINE/	17613
232	Uzbekistan.mp. or exp UZBEKISTAN/	2271
233	Vanuatu.mp. or exp VANUATU/	708
234	Venezuela.mp. or exp VENEZUELA/	7125
235	exp VIETNAM/ or Vietnam.mp.	18647
236	(West Bank and Gaza).mp.	220
237	Republic of Yemen.mp. or exp Yemen/	1396
238	Zambia.mp. or exp ZAMBIA/	6294
239	Zimbabwe.mp. or exp ZIMBABWE/	7682
240	Guatemala.mp. or exp GUATEMALA/	4450
241	Guinea.mp. or exp GUINEA-BISSAU/ or exp EQUATORIAL GUINEA/ or exp GUINEA/ or exp PAPUA NEW/	162475
242	kabul.mp.	452
243	Porto-Novo.mp.	34
244	Hogbonou.mp.	0
245	adjace.mp.	0

246	cotonou.mp.	382
247	kutonu.mp.	0
248	ouagadougou.mp.	692
249	ouaga.mp.	4
250	bujumbura.mp	71
251	usumbura.mp.	3
252	phnom penh.mp.	399
253	bangui.mp.	306
254	bangi.mp.	27
255	n'djamena.mp.	111
256	ndjamena.mp.	111
257	fort lamy.mp.	23
258	moroni.mp.	26
259	kinshasa.mp.	1010
260	asmara.mp.	73
261	asmera.mp.	0
262	addis ababa.mp.	1892
263	addis abeba.mp.	82
264	banjul.mp.	84
265	bathurst.mp.	58
266	conakry.mp	297

267	bissau.mp.	1267
268	port-au-prince.mp.	320
269	pyongyang.mp.	31
270	monrovia.mp.	430
271	antananarivo.mp.	320
272	tananarive.mp.	83
273	tana.mp.	180
274	lilongwe.mp.	464
275	bamako.mp.	773
276	maputo.mp	543
277	lourenco.mp.	117
278	marques.mp.	476
279	kathmandu.mp.	1612
280	niamey.mp.	289
281	kigali.mp	428
282	freetown.mp	167
283	free-town.mp	6
284	mogadishu.mp	90
285	xamar.mp	0
286	hamar.mp	36
287	muqdisho.mp	0
288	maqadishu.mp	0

289	juba.mp	82
290	dodoma.mp	107
291	dar es salaam.mp	1606
292	lome.mo	498
293	kampala.mp	1739
294	harare.mp	1131
295	salisbury.mp	465
296	yerevan.mp	112
297	dhaka.mp	2341
298	dacca.mp	122
299	thimphu.mp	0
300	sucre.mp	1093
301	charcas.mp	2
302	la plata.mp	564
303	chuquisaca.mp	26
304	la paz.mp	761
305	prai.mp	47
306	yaounde.mp	1204
307	jaunde.mp	0
308	brazzaville.mp	615
309	yamoussoukro.mp	21

310	cario.mp	2717
311	accra.mp	1393
312	tegucigalpa.mp	81
313	tegus.mp	26
314	new delhi.mp	3169
315	jakarta.mp	1125
316	nairobe.mp	2843
317	south tarawa.mp	14
318	tarawa.mp	36
319	teinainano.mp	0
320	pristina.mp	73
321	prishtina.mp	75
322	bishkek.mp	60
323	pishpek.m	0
324	frunze.mp	95
325	vietiane.mp	302
326	maseru.mp	48
327	nouakchott.mp	96
328	palikir.mp	0
329	chisinau.mp	21
330	kishinev.mp	38
331	rabat.mp	150

332	nay pyi taw.mp	8
333	naypyidaw.mp	0
334	nepranytau.mp	0
335	naypyitaw.mp	1
336	kyetpyay.mp	0
337	pyinmana.mp	4
338	kyatpyay.mp	0
339	pyinmana.mp	4
340	yangon.mp	272
341	rangoon.mp	155
342	managua.mp	185
343	abuja.mp	441
344	lagos.mp	2781
345	islamabad.mp	1065
346	port moresby.mp	364
347	moresby.mp	365
348	pom town.mp	0
349	manila.mp	1240
350	apia.mp	74
351	dakar.mp	2196
352	honiara.mp	55

353	jayawardenepura.mp	4
354	jayewardenepura.mp	13
355	khartoum.mp	1027
356	mbabane.mp	29
357	embabane.mp	0
358	lobamba.mp	0
359	damascus.mp	286
360	dushanbe.mp	69
361	dyushambe.mp	1
362	stalinabad.mp	2
363	dili.mp	1444
364	kyiv.mp	217
365	kiev.mp	771
366	tashkent.mp	248
367	toshkent.mp	1
368	port vila.mp	19
369	hanoi.mp	1355
370	ha noi.mp	46
371	sana'a.mp	247
372	sanaa.mp	247
373	sana.mp	427
374	lusaka.mp	1091

375	ulaanbaatar.mp	247
376	ulan-bator.mp	34
377	luanda.mp	228
378	tbilisi.mp	413
379	amman.mp	702
**380**	**88 or 89 or 90 or 91 or 92 or 93 or 94 or 95 or 96 or 97 or 98 or 99 or 100 or 101 or 102 or 103 or 104 or 105 or 106 or 107 or 108 or 109 or 110 or 111 or 112 or 113 or 114 or 115 or 116 or 117 or 118 or 119 or 120 or 121 or 122 or 123 or 124 or 125 or 126 or 127 or 128 or 129 or 130 or 131 or 132 or 133 or 134 or 135 or 136 or 137 or 138 or 139 or 140 or 141 or 142 or 143 or 144 or 145 or 146 or 147 or 148 or 149 or 150 or 151 or 152 or 153 or 154 or 155 or 156 or 157 or 158 or 159 or 160 or 161 or 162 or 163 or 164 or 165 or 166 or 167 or 168 or 169 or 170 or 171 or 172 or 173 or 174 or 175 or 176 or 177 or 178 or 179 or 180 or 181 or 182 or 183 or 184 or 185 or 186 or 187 or 188 or 189 or 190 or 191 or 192 or 193 or 194 or 195 or 196 or 197 or 198 or 199 or 200 or 201 or 202 or 203 or 204 or 205 or 206 or 207 or 208 or 209 or 210 or 211 or 212 or 213 or 214 or 215 or 216 or 217 or 218 or 219 or 220 or 221 or 222 or 223 or 224 or 225 or 226 or 227 or 228 or 229 or 230 or 231 or 232 or 233 or 234 or 235 or 236 or 237 or 238 or 239 or 240 or 241 or 242 or 243 or 244 or 245 or 246 or 247 or 248 or 249 or 250 or 251 or 252 or 253 or 254 or 255 or 256 or 257 or 258 or 259 or 260 or 261 or 262 or 263 or 264 or 265 or 266 or 267 or 268 or 269 or 270 or 271 or 272 or 273 or 274 or 275 or 276 or 277 or 278 or 279 or 280 or 281 or 282 or 283 or 284 or 285 or 286 or 287 or 288 or 289 or 290 or 291 or 292 or 293 or 294 or 295 or 296 or 297 or 298 or 299 or 300 or 301 or 302 or 305 or 306 or 307 or 308 or 309 or 310 or 311 or 312 or 313 or 314 or 315 or 316 or 317 or 318 or 319 or 320 or 321 or 322 or 323 or 324 or 325 or 326 or 327 or 328 or 329 or 330 or 331 or 332 or 333 or 334 or 335 or 336 or 337 or 338 or 339 or 340 or 341 or 342 or 343 or 344 or 345 or 346 or 347 or 348 or 349 or 350 or 351 or 352 or 353 or 354 or 355 or 356 or 357 or 358 or 359 or 360 or 361 or 362 or 363 or 364 or 365 or 366 or 367 or 368 or 369 or 370 or 371 or 372 or 373 or 374 or 375 or 376 or 377 or 378 or 379**	**1600818**
381	11 AND 87 AND 380	246
