# Supplementary material for: Cost-effectiveness of neonatal surgery for congenital anomalies in low-income and middle-income countries: a systematic review protocol
Source: BMJ Paediatr Open. 2020 Aug 30;4(1):e000755. doi: 10.1136/bmjpo-2020-000755 (PMC7462241; doi:10.1136/bmjpo-2020-000755)
Supplement: Author's manuscript [file bmjpo-2020-000755.draft_revisions.pdf]

BMJ Paediatrics Open

BMJ Paediatrics Open is committed to open peer review. As part of this commitment we make the peer review history of every article we publish publicly available.

When an article is published we post the peer reviewers' comments and the authors' responses online. We also post the versions of the paper that were used during peer review. These are the versions that the peer review comments apply to.

The versions of the paper that follow are the versions that were submitted during the peer review process. They are not the versions of record or the final published versions. They should not be cited or distributed as the published version of this manuscript.

BMJ Paediatrics Open is an open access journal and the full, final, typeset and author-corrected version of record of the manuscript is available on our site with no access controls, subscription charges or pay-per-view fees (http://bmjpaedsopen.bmj.com).

If you have any questions on BMJ Paediatrics Open's open peer review process please email info.bmjpo@bmj.com

BMJ Paediatrics Open

Cost-Effectiveness of Neonatal Surgery for Congenital Anomalies in Low- and Middle-Income Countries: A Systematic Review Protocol

Journal:	*BMJ Paediatrics Open*
Manuscript ID	bmjpo-2020-000755
Article Type:	Protocol
Date Submitted by the Author:	09-Jun-2020
Complete List of Authors:	Kim, Na Eun; Boston Medical Center; King's College London<br>Vervoot, Dominique; Johns Hopkins Bloomberg School of Public Health<br>Hammouri, Ahmad; Al Quds University<br>Riboni, Cristiana; University of Pavia<br>Salem, Hosni; Cairo University<br>Grimes, Caris; King's College London; Medway NHS Foundation Trust, Department of Surgery<br>Wright, Naomi; King's Centre for Global Health and Health Partnerships
Keywords:	Neonatology

SCHOLARONE™
Manuscripts

I, the Submitting Author has the right to grant and does grant on behalf of all authors of the Work (as defined in the below author licence), an exclusive licence and/or a non-exclusive licence for contributions from authors who are: i) UK Crown employees; ii) where BMJ has agreed a CC-BY licence shall apply, and/or iii) in accordance with the terms applicable for US Federal Government officers or employees acting as part of their official duties; on a worldwide, perpetual, irrevocable, royalty-free basis to BMJ Publishing Group Ltd ("BMJ") its licensees and where the relevant Journal is co-owned by BMJ to the co-owners of the Journal, to publish the Work in this journal and any other BMJ products and to exploit all rights, as set out in our licence.

The Submitting Author accepts and understands that any supply made under these terms is made by BMJ to the Submitting Author unless you are acting as an employee on behalf of your employer or a postgraduate student of an affiliated institution which is paying any applicable article publishing charge ("APC") for Open Access articles. Where the Submitting Author wishes to make the Work available on an Open Access basis (and intends to pay the relevant APC), the terms of reuse of such Open Access shall be governed by a Creative Commons licence – details of these licences and which Creative Commons licence will apply to this Work are set out in our licence referred to above.

Other than as permitted in any relevant BMJ Author's Self Archiving Policies, I confirm this Work has not been accepted for publication elsewhere, is not being considered for publication elsewhere and does not duplicate material already published. I confirm all authors consent to publication of this Work and authorise the granting of this licence.

Confidential: For Review Only

Cost-Effectiveness of Neonatal Surgery for Congenital Anomalies in Low- and Middle-Income Countries: A Systematic Review Protocol

Authors:
Na Eun Kim, MD
Boston Medical Center
88 East Newton St C515, Boston, Massachusetts, United States
Naeun88@gmail.com
(Corresponding author)

Dominique Vervoort, MD
Johns Hopkins Bloomberg School of Public Health
615 North Wolfe Street, 21205 Baltimore, Maryland, United States
vervoortdominique@hotmail.com

Ahmad Hammouri, MD
Al-Quds University Medical School
Al-Salam Street, Hebron, West-Bank, Palestine
ahmadalhammouri94@gmail.com

Cristiana Riboni
University of Pavia
C.so Strada Nuova, 65 - Pavia, Italy
cristiana.riboni01@universitadipavia.it

Hosni Salem, MD
Faculty of Medicine, Cairo University
Cairo, Egypt

Caris E Grimes
King's Centre for Global Health and Health Partnerships,
School of Population Health and Environmental Sciences,
Faculty of Life Sciences and Medicine, King's College London, London, UK.
and Department of Surgery, Medway NHS Foundation Trust, Windmill Road, Gillingham, Kent ME7 5NY. UK
Caris.grimes@kcl.ac.uk

Naomi Wright
King's Centre for Global Health and Health Partnerships,
School of Population Health and Environmental Sciences,
Faculty of Life Sciences and Medicine, King's College London, London, UK.
naomiwright@doctors.org.uk

Confidential: For Review Only

Amendments:
Version 1

PROSPERO registration number
CRD 42020172971

Keywords: Neonatal Surgery, Paediatric Surgery, Congenital Anomalies, Cost-effectiveness, LMICs

Abstract:

Introduction

Congenital anomalies are the 5th leading cause of death in under 5-year olds globally (591,000 deaths reported, 2016). Over 95% of the deaths occur in low- and middle-income countries (LMICs). Two-thirds of the congenital anomaly health burden could be averted through surgical intervention and such interventions have been found to be cost-effective. This systematic review aims to evaluate the cost-effectiveness of neonatal surgery for congenital anomalies in LMICs.

Methods and analysis

A systematic literature review will be conducted in PubMed, MEDLINE, Embase, and Cochrane Library databases for articles on the cost-effectiveness of neonatal surgery for congenital anomalies in LMICs. The following search strings will be used: 1. Congenital anomalies; 2. LMICs; 3. Cost-effectiveness of surgical interventions. Articles will be uploaded to Covidence software, duplicates removed and remaining articles screened by two independent reviewers. Cost information for interventions or procedures will be extracted by country and condition. Outcome measurements by reported unit and cost-effectiveness ratios will be extracted. Methodological quality of each article will be assessed using the Drummond checklist for economic evaluations.

Ethics and dissemination

No ethical approval is required for conducting the systematic review. There will be no direct collection of data from individuals. The finalized article will be published in a scientific journal for dissemination. The protocol has been registered with PROSPERO.

Conclusion

Congenital anomalies form a large component of the global health burden that is amenable to surgical intervention. This study will systematically review the current literature on the cost-effectiveness of neonatal surgery for congenital anomalies in LMICs.

Confidential: For Review Only

What is known about the subject:

- Congenital anomalies are a large component of the global health burden, recently rising to become the 5th leading cause of death in children under 5 years globally.
- Over 95% of deaths occur in low and middle income countries (LMICs).
- Surgical interventions have been shown to be cost-effective in reducing the burden of disease for some congenital anomalies.

What this study hopes to add:

- To systematically investigate the cost-effectiveness of neonatal surgery for the management of congenital anomalies in LMICs.
- To provide an evidence-based summary of the cost-effectiveness of neonatal surgery for congenital anomalies in LMICs to direct future interventions and investments.

Confidential: For Review Only

Manuscript:

Introduction:

Congenital anomalies constitute a large global health burden, accounting for an estimated 591,000 deaths worldwide in 2016 [1] . The burden of disease from congenital anomalies falls most heavily on low- and middle-income countries (LMICs), where over 95% of the deaths occur [2, 3]. The burden of disease, traditionally expressed in disability-adjusted life years (DALYs), represents a health gap in a population due to early mortality and years of healthy life lost. As an example of this health gap, an estimated 140,154 DALYs are potential avertable by neonatal surgery in Uganda, however only 3.5% of the need is currently being met by the health system [4]. The higher burden in LMICs may also be attributed to a higher disease incidence related to a higher rate of micronutrient and macronutrient deficiencies, exposure to teratogens, intrauterine infections, and self-medication. In addition, elective pregnancy termination following prenatal diagnosis is less available in LMICs compared to high-income countries (HICs) [5].

Mortality rates for many congenital anomalies in LMICs are high. For example, gastroschisis mortality has been reported as 75-100% in many paediatric surgical centres across sub-Saharan Africa compared to less than 4% in HICs [5, 6, 7]. Disturbingly, published high mortality rates from congenital anomalies may even be an underestimate of the true burden of disease due to sizable hidden mortality. Children that do not reach health facilities and die at home or in transit are traditionally not accounted for [8]. EUROCAT national estimates show that infant mortality from congenital anomalies could be up to 29% higher than that reported by the World Health Organization (WHO) [9]. Furthermore, it is difficult to estimate the true burden of congenital anomalies in LMICs due to a lack of congenital anomaly registries and deficient research.

Traditionally, surgical care for neonates has been perceived as too complex and expensive to be prioritized or even implemented in LMICs, amidst competing public and global health priorities. However, a growing body of literature has arisen evaluating the potential of surgical interventions to reduce the burden of disease due to congenital anomalies in LMICs [10, 11, 12, 13]. 57% of DALYs caused by cleft lip and palate, congenital heart anomalies, and neural tube defects are potentially amenable to surgical care in LMICs [10]. Among gastroschisis patients in Uganda, up to 58.7 DALYs could be avertable per patient if appropriate surgical care was available [14]. Despite this high unmet need that can be prevented with surgical care, the burden of surgical disease in the pediatric population remains high in LMICs. Many surgical conditions are congenital, yet surgical care in LMICs is often delayed, worsening outcomes due to presenting with more advanced disease processes [11, 15]. In Kenya, only 13.5% of the required surgical interventions were performed for common congenital conditions requiring surgical care [13].

Surgical care has been found to be cost-effective in LMICs across various fields, including pediatric surgery [16, 17]. Treating congenital anomalies such as cleft lip and palate, anorectal malformation, congenital diaphragmatic hernia, and congenital heart defects can have significant economic impact by adding to lifetime individual income and quality life lived [12, 18, 19, 20] Conversely, untreated conditions such as hydrocephalus in infants can exact a large economic burden, whereas treating them has been shown to have a favourable cost-benefit ratio [21].

It is important to fully understand the economic burden of congenital anomalies and the cost-effectiveness of surgical interventions to improve outcomes. This study aims to systematically review existing literature on the cost-effectiveness of neonatal surgery for the management of congenital anomalies in LMICs.

Aim: To conduct a systematic review that identifies and analyzes cost-effectiveness of neonatal surgical interventions for congenital anomalies in LMICs.

Objectives:

1. To systematically identify studies on cost-effectiveness of neonatal surgery for congenital anomalies in LMICs;
2. Evaluate the cost-effectiveness of neonatal surgery for congenital anomalies in LMICs;
3. To provide an evidence-based summary of cost-effectiveness of neonatal surgery for congenital anomalies in LMICs to direct future interventions and investments in neonatal surgery;
4. Critically appraise the quality of the studies included in the systematic review.

Confidential: For Review Only

Methods and analysis

A systematic literature review will be conducted following the PRISMA-P guidelines (Supplementary file 1). Any amendments to the protocol will be reported in the publication of the results.

Inclusion/ Exclusion criteria:

Inclusion criteria:

Conditions: Abdominal Wall Defect, Aganglionosis, Anal Atresia, Anorectal Malformation, Anorectal Stenosis, Apple Peel Syndrome, Biliary Atresia, Birth Defects, Bladder Exstrophy, Branchial Tag, Branchial Vestige, Bronchopulmonary Sequestration, Cervicoaural Fistula, Choledochal Cyst, Cleft Lip, Cleft Palate, Clubfoot, Colonic Atresia, Congenital Abnormalities, Congenital Anomalies, Congenital Cystic Adenomatoid Malformation of Lung, Congenital Diaphragmatic Hernia, Congenital Heart Defects, Congenital Hydronephrosis, Congenital Malformation, Conjoined Twins, Cryptorchidism, Diaphragmatic Eventration, Down Syndrome, Duodenal Atresia, Duodenal Obstruction, Duodenal Web, Epispadias, Exomphalos, Fetal Malformation, Gastroschisis, Hirschsprung's Disease, Hydrocele, Hypospadias, Ileal Atresia, Imperforate Anus, Imperforate Hymen, Indeterminate Sex, Intestinal Atresia, Jejunal Atresia, Jejuno-Ileal Atresia, Malrotation, eural Tube Defects, Oesophageal Atresia, Omphalocele, Orofacial Clefts, Pectus Excavatum, Pes Cavus, Phimosis, Polycystic Kidney Disease, Polydactyly, Preauricular Sinus, Redundant Neck Fold, Spina Bifida, Syndactyly, Tongue Tie, Tracheo Oesophageal Fistula, Umbilical Hernia, Undescended Testicle, Volvulus, Webbed Neck

Age: Neonatal

Place: Low- and middle-income countries (as defined by World Bank 2019 Classification)

Intervention: Surgical or operative interventions

Exclusion criteria:

Conditions: All conditions not listed in inclusion criteria

Age: Greater than 28 days of life

Place: High-income countries.

Intervention: Any procedure performed antenatally or after the first 28 days of life will be excluded.

Search strategy:

A systematic literature search will be conducted using the following search strings: 1. Congenital anomalies; 2. LMICs; 3. Cost-effectiveness of surgical interventions (Table 1). The following databases will be used: PubMed, MEDLINE, EMBASE, and Cochrane Library. The search will include English, French, Spanish, Italian, and Arabic articles and will be restricted to human studies. There will be no restrictions on publication date or study design.

Study design

Published, peer-reviewed journal articles will be included. Any study without explicit cost data or health outcomes data will be excluded. Case reports, editorials, letters to the editor, and literature reviews will be excluded. Abstracts without available full text will be excluded.

Methodological quality

The studies will be assessed for their methodological quality of economic evaluations using the Drummond 10-point checklist [22] (Table 2). The results will be summarized in a table format in the results section.

Study screening

Articles will be uploaded to Covidence software (Melbourne, Australia), duplicate articles will be removed, and remaining articles will be screened by two independent reviewers. All potential eligible articles will be screened in full

Confidential: For Review Only

text for final selection by two independent reviewers. The reference lists of the included articles will be screened to identify further eligible studies. Conflicts between the two reviewers' assessments will be resolved by a consensus meeting of all authors.

Data extraction

The following data will be extracted: study type or design, study population, study period, country, year of publication, journal, author name(s), number of patients, patient demographics, type of condition, type of intervention, gestational age, weight, time from birth to presentation, costs incurred during treatment, outcome of intervention (mortality and morbidity which will be reported using the Clavien-Dindo scoring system) [23].

Main outcomes will be the cost of surgical interventions and health outcomes. The effectiveness method of DALYs, HALYs, and QALYs will be extracted. Reported incremental cost-effectiveness ratio and/or potential gains in life expectancy will be extracted. Data reported for different countries or procedures will be extracted as separate results.

Data synthesis

The data will be organized into: author(s), the year of publication, intervention or procedure, country or site of intervention, gross domestic product, costs per outcome, outcome unit, and currency.

Cost data will be organized into cost to the provider and cost to the patient. The cost will be converted to USD, calculated by the currency year. Quantitative analysis will be undertaken by calculating the median values and interquartile range for the intervention or procedures. Meta-analysis will likely not be feasible due to the limited availability of data. If our search results in the appropriate data a meta-analysis will be conducted. Appropriate data is defined as two or more homogeneous studies comparing the cost and outcomes of a specific surgical procedure to another non-surgical intervention. The meta-analysis will be conducted in Stata.

The GRADE (Grading of Recommendations Assessment, Development, and Evaluation) system will be used to rate the quality of evidence and the strength of recommendations.

Patient and public involvement

Whilst we recognise the importance of public and patient involvement in research, on this occasion it has not been feasible to incorporate this into the design of the study due to difficulty engaging with patients and their families affected by congenital anomalies within LMICs in such a project. We will endeavour to ensure that a summary of the results of the study is provided in lay language and disseminated for public viewing.

Ethics and Dissemination

No ethical approval was required for conducting the systematic review. There was no direct collection of data from individuals. The finalized article will be published in a scientific journal for dissemination.

Limitations

The studies may have been conducted in different settings, such as government facilities, non-profit hospitals, and surgical mission trips, which may limit direct comparisons. Costs may also be reported that vary in the components included. In addition, the health outcome calculations will be different by study, which may or may not include age weighting or discounting. The search engines used may not identify all articles. Lastly, it is unlikely that the incremental cost-effectiveness ratio will be directly comparable among studies as the alternative comparison may be different for each study.

Discussion:

Congenital anomalies are reported to account for approximately 62.9 million or 2.4% of global DALYs in 2016 [24]. Published literature from LMICs commonly describe the burden of disease from congenital disease in terms of mortality instead of DALYs, emphasizing the difference in mortality rates compared to those from HICs. Reducing infant mortality due to congenital anomalies will help to meet the Sustainable Development Goals (SDG) aim to reduce preventable deaths in neonates to as low as 12 per 1,000 live births by 2030 [30]. Current literature has shown that surgical care for congenital anomalies can reduce the burden of disease in a cost-effective manner [12, 17, 18, 19, 31].

Confidential: For Review Only

An estimated two-thirds of the burden of disease related to congenital anomalies can be averted through surgical care [32]. Despite this potential, the burden of surgical disease in this neonatal population remains high in LMICs.

There is a growing body of literature for cost effectiveness of surgical interventions for congenital anomalies but to our knowledge there has been no systematic analysis consolidating these studies. The most recent literature review by *Stolk et al* was completed in 2000 reporting two complete economic evaluations of cost-effectiveness in neonatal surgery for congenital anomalies which were not done in the context of LMICs [31, 33, 34]. This project will provide a crucial addition to the literature by providing a systematic review on current literature of cost effectiveness analysis of neonatal surgery for congenital anomalies in LMICs.

Confidential: For Review Only

References

1. WHO 2016. Disease burden and mortality estimates. https://www.who.int/healthinfo/global_burden_disease/estimates/en/ (Accessed 6 March 2020).
2. Sitkin NA, Farmer DL. Congenital anomalies in the context of global surgery. Semin Pediatr Surg. 2016 Feb;25(1):15-8. doi: 10.1053/j.sempedsurg.2015.09.004. Epub 2015 Sep 16.
3. WHO 2012. Mortality Statistics. http:// apps. who. int/ healthinfo/ statistics/ mortality/who. htm (accessed 7 July 2019).
4. Badrinath R, Kakembo N, Kisa P, et al. Outcomes and unmet need for neonatal surgery in a resource-limited environment: estimates of global health disparities from Kampala, Uganda. J Pediatr Surg. 2014 Dec;49(12):1825-30. doi: 10.1016/j.jpedsurg.2014.09.031. Epub 2014 Oct 1.
5. Sitkin NA, Ozgediz D, Donkor P, et al. Congenital anomalies in low- and middle income countries: the unborn child of global surgery. World J Surg 2015;39:36–40.
6. Wright NJ, Zani A, Ade-Ajayi N. Epidemiology, management and outcome of gastroschisis in Sub-Saharan Africa: Results of an international survey. *Afr J Paediatr Surg* 2015; **12**(1): 1-6.
7. Bradnock T, Marven S, Owen A, et al. Gastroschisis: one year outomes from national cohort study. *BMJ* 2011; **343**(d6749).
8. Modell B, Berry RJ, Boyle CA, et al. Global regional and national causes of child mortality. Lancet 2012;380:1556.
9. Boyle B, Addor MC, Arriola L, et al. Estimating Global Burden of Disease due to congenital anomaly: an analysis of European data. *Arch Dis Child Fetal Neonatal Ed*. 2018;103(1):F22-F28. doi:10.1136/archdischild-2016-311845
10. Higashi, H., J. J. Barendregt, and T. Vos. 2013. "The Burden of Congenital Anomalies Amenable to Surgeries in Low-Income and Middle-Income Countries: A Modeled Analysis." The Lancet 381: S62.
11. Ozgediz, D., and D. Poenaru. 2012. "The Burden of Pediatric Surgical Conditions in Low and Middle Income Countries: A Call to Action." Journal of Pediatric Surgery 47 (12): 2305–11.
12. Corlew, D. 2010. "Estimation of Impact of Surgical Disease through Economic Modeling of Cleft Lip and Palate Care." World Journal of Surgery 34 (3): 391–96.
13. Wu V, Poenaru D. 2013. Burden of surgically correctable disabilities among children in the Dadaab refugee camp. World J Surg 37:1536–1543. doi:10.1007/s00268-012-1899-z
14. Ford K, Poenaru D, Moulot O, et al. Gastroschisis: Bellwether for neonatal surgery capacity in low resource settings? J Pediatr Surg. 2016 Aug;51(8):1262-7. doi: 10.1016/j.jpedsurg.2016.02.090. Epub 2016 Mar 12.
15. Poenaru, D., J. Pemberton, C. Frankfurter, et al. 2013. "Establishing Disability Weights for Congenital Pediatric Surgical Disease: A Multi-modal Study." The Lancet 381: S3115
16. Chao TE, Sharma K, Mandigo M, et al. Cost-effectiveness of surgery and its policy implications for global health: a systematic review and analysis. *Lancet Glob Health*. 2014;2(6):e334-e345. doi:10.1016/S2214-109X(14)70213-X
17. Grimes CE, Henry JA, Maraka J, et al. Cost-effectiveness of surgery in low- and middle-income countries: a systematic review. World J Surg. 2014 Jan;38(1):252-63. doi: 10.1007/s00268-013-2243-y.
18. Poley MJ, Brouwer WB, Busschbach JJ, et al. Cost-effectiveness of neonatal surgery: first greeted with scepticism, now increasingly accepted. Pediatr Surg Int. 2008 Feb;24(2):119-27. Epub 2007 Nov 6.
19. Cardarelli M, Vaikunth S, Mills K, et al. Cost-effectiveness of Humanitarian Pediatric Cardiac Surgery Programs in Low and Middle Income Countries. JAMA Netw Open. 2018 Nov 2;1(7):e184707. doi: 10.1001/jamanetworkopen.2018.4707.

20. Poley MJ, Stolk EA, Langemeijer RA, Molenaar JC, Busschbach JJ. The cost-effectiveness of neonatal surgery and subsequent treatment for congenital anorectal malformations. *J Pediatr Surg*. 2001;36(10):1471-1478. doi:10.1053/jpsu.2001.27025
21. Warf, B. C., B. C. Alkire, S. Bhai, et al. 2011. "Costs and Benefits of Neurosurgical Intervention for Infant Hydrocephalus in Sub-Saharan Africa." Journal of Neurosurgical Pediatrics 8 (5): 509–21.
22. Drummond, M. F., Sculpher, Mark J, Claxton, Karl, et al. Methods for the Economic Evaluation of Health Care Programmes. 4th ed. Oxford: OUP Oxford, 2015. Web.
23. Dindo D, Demartines N, Clavien PA. Classification of surgical complications: a new proposal with evaluation in a cohort of 6336 patients and results of a survey. Ann Surg. 2004;240(2):205–13.
24. Global Health Estimates 2016: Disease burden by Cause, Age, Sex, by Country and by Region, 2000-2016. Geneva, World Health Organization
25. Manson J, Ameh E, Canvassar N, et al. Gastroschisis: a multi-centre comparison of management and outcome. Afr J Paediatr Surg. 2012 Jan-Apr;9(1):17-21. doi: 10.4103/0189-6725.93296.
26. Du L, Pan WH, Cai W, et al. Delivery room surgery: an applicable therapeutic strategy for gastroschisis in developing countries. World J Pediatr. 2014 Feb;10(1):69-73. doi: 10.1007/s12519-014-0455-3. Epub 2014 Jan 25.
27. Ameh EA, Chirdan LB. Ruptured exomphalos and gastroschisis: a retrospective analysis of morbidity and mortality in Nigerian children. Pediatr Surg Int. 2000;16(1-2):23-5.
28. Askarpour S, Ostadian N, Javaherizadeh H, et al. Omphalocele, gastroschisis: epidemiology, survival, and mortality in Imam Khomeini hospital, Ahvaz-Iran. Pol Przegl Chir. 2012 Feb;84(2):82-5. doi: 10.2478/v10035-012-0013-4.
29. Sekabira J, Hadley GP. Gastroschisis: a third world perspective. Pediatr Surg Int. 2009 Apr;25(4):327-9. doi: 10.1007/s00383-009-2348-4. Epub 2009 Mar 14.
30. United Nations. Sustainable Development Goals. 2015 ht tps://sustainabledevelopment. un. org/ ? me nu= 1300 (accessed 26 Jan 2016).
31. Stolk EA, Post HA, Rutten FF, et al. Cost-effectiveness of neonatal surgery: a review. J Pediatr Surg. 2000 Apr;35(4):588-92.
32. Debas HT, Donkor P, Gawande A, et al. Disease control priorities, 3rd edn—volume 1: essential surgery. Washington, DC: World Bank, 2015.
33. Roberts TE. Economic evaluation and randomised controlled trial of extracorporeal membrane oxygenation: UK collaborative trial. The Extracorporeal Membrane Oxygenation Economics Working Group. BMJ. 1998 Oct 3;317(7163):911-5; discussion 915-6.
34. Hackam DJ, Superina RA, Pearl RH. Single-stage repair of Hirschsprung's disease: A comparison of 109 patients over 5 year. J Pediatr Surg 32:1028-1031, 1997

Supplementary files
Supplementary file 1: Prisma checklist
Supplementary file 2: Example Search Strategy

Authors' contributions
All authors contributed to the design of the systematic review and drafted the manuscript including its revisions. All authors gave final approval of the version to be published and agree to be accountable for all aspects of the work and ensure that questions related to the accuracy of the work are appropriately investigated and resolved.

Funding statement:
Naomi Wright receives funding from the Wellcome Trust to undertake a Clinical PhD in Global Health at King's Centre for Global Health and Health Partnerships, King's College London (Funder Reference: 203905/Z/16/Z).
No financial relationships

Competing interests:
There are no conflicts of interests.

Confidential: For Review Only

Tables:

Table 1: Search Strategy

The following search strings were used during the literature search for this systematic review (Table 1). Search string 1 encompasses congenital anomalies found in neonates that commonly require surgical intervention. Search string 2 includes all low- and middle- income countries. Search string 3 describes the cost-effectiveness of an intervention.

Search String 1	*Search String 2*	*Search String 3*
Congenital Abnormalities, Congenital Anomalies, Congenital Malformation, Abdominal Wall Defect, Aganglionosis, Anal Atresia, Anal Stenosis, Anorectal Malformation, Anorectal Stenosis, Apple Peel Syndrome, Biliary Atresia, Birth Defects, Bladder Exstrophy, Branchial Tag, Branchial Vestige, Bronchopulmonary Sequestration, Bronchogenic Cyst, Cervicoaural Fistula, Choledochal Cyst, Cleft Lip, Cleft Palate, Clubfoot, Colonic Atresia, Congenital Cystic Adenomatoid Malformation of Lung, Congenital Diaphragmatic Hernia, Congenital Heart Defects, Congenital Hydronephrosis, Conjoined Twins, Cryptorchidism, Diaphragmatic Eventration, Down Syndrome, Duodenal Atresia, Duodenal Obstruction, Duodenal Web, Epispadias, Exomphalos, Fetal Malformation, Gastroschisis, Hirschsprung's Disease, Hydrocele, Hypospadias, Ileal Atresia, Imperforate Anus, Imperforate Hymen, Indeterminate Sex, Intestinal Atresia, Jejunal Atresia, Jejuno-Ileal Atresia, Malrotation, Maxillofacial Abnormalities, Megacolon, Mouth Abnormalities, Neural Tube Defects, Oesophageal Atresia, Omphalocele, Orofacial Clefts, Pectus Excavatum, Pes Cavus, Phimosis, Polycystic Kidney Disease, Polydactyly, Preauricular Sinus,	LMICs, Low- and Middle-Income Countries, Developing Countries, Low Resource Settings, Underdeveloped Countries, Low-Income Countries, Middle-Income Countries, Limited Resource Settings, Africa South of the Sahara, Sub-Saharan Africa, Less Resourced Communities, Afghanistan, Albania, Algeria, American Samoa, Angola, Argentina, Armenia, Azerbaijan, Bangladesh, Belarus, Belize, Benin, Bhutan, Bolivia, Bosnia and Herzegovina, Botswana, Brazil, Bulgaria, Burkina Faso, Burundi, Cabo Verde, Cambodia, Cameroon, Central African Republic, Chad, China, Colombia, Comoros, Democratic Republic of the Congo, DRC, Republic of the Congo, Costa Rica, Cote d'Ivoire, Ivory Coast, Cuba, Djibouti, Dominica, Dominican Republic, Ecuador, Egypt, Egypt Arab Republic, El Salvador, Equatorial Guinea, Eritrea, Eswatini, Ethiopia, Fiji, Gabon, Gambia, Georgia, Ghana, Grenada, Guatemala, Guinea, Guinea-Bissau, Guyana, Haiti, Honduras, India, Indonesia, Islamic Republic of Iran, Iraq, Jamaica, Jordan, Kazakhstan, Kenya, Kiribati, Democratic People's Republic of Korea, Kosovo, Kyrgyz Republic, Lao	Cost, Cost Effectiveness, Cost Benefit Analysis, Cost of Illness, Cost Effective Surgical Intervention, Health Care Cost, DALYs, Disability Adjusted Life Years, QALYs, Quality Adjusted Life Years, HALYs, Health Adjusted Life Years

Confidential: For Review Only

Rectosigmoid Aganglionosis, Redundant Neck Fold, Renal Anomalies, Spina Bifida, Syndactyly, Tongue Tie, Tracheo-Oesophageal Fistula, Umbilical Hernia, Undescended Testicle, Urogenital Abnormalities, Volvulus, Webbed Neck	PDR, Laos, Lebanon, Lesotho, Liberia, Libya, Madagascar, Malawi, Malaysia, Maldives, Mali, Marshall Islands, Mauritania, Mauritius, Mexico, Micronesia, Moldova, Mongolia, Montenegro, Morocco, Mozambique, Myanmar, Namibia, Nauru, Nepal, Nicaragua, Niger, Nigeria, North Macedonia, Pakistan, Papua New Guinea, Paraguay, Peru, Philippines, Romania, Russian Federation, Rwanda, Samoa, Sao Tome and Principe, Senegal, Serbia, Sierra Leone, Solomon Islands, Somalia, South Africa, South Sudan, Sri Lanka, Saint Lucia, Saint Vincent and the Grenadines, Sudan, Suriname, Swaziland, Syrian Arab Republic, Tajikistan, Tanzania, Thailand, Timor-Leste, Togo, Tonga, Tunisia, Turkey, Turkmenistan, Tuvalu, Uganda, Ukraine, Uzbekistan, Vanuatu, Venezuela, Vietnam, West Bank and Gaza, Republic of Yemen, Zambia, Zimbabwe	

Confidential: For Review Only

Table 2: Drummond Checklist

Item		Yes (Page number)	No	Not Clear	Not Appropriate
**Study Design**					
1	The research question is stated				
2	The economic importance of the research question is stated				
3	The viewpoint(s) of the analysis are clearly stated and justified				
4	The rationale for choosing alternative programmes or interventions compared is stated				
5	The alternatives being compared are clearly described				
6	The form of economic evaluation used is stated				
7	The choice of form of economic evaluation is justified in relation to the questions addressed				
**Data Collection**					
8	The source(s) of effectiveness estimates used are stated				
9	Details of the design and results of effectiveness study are given (if based on a single study)				
10	Details of the methods of synthesis or meta-analysis of estimates are given (if based on a synthesis of a number of effectiveness studies)				
11	The primary outcome measure(s) for the economic evaluation are clearly stated				
12	Methods to value benefits are stated				
13	Details of the subjects from whom valuations were obtained were given				
14	Productivity changes (if included) are reported separately				
15	The relevance of productivity changes to the study question is discussed				
16	Quantities of resource use are reported separately from their unit costs				
17	Methods for the estimation of quantities and unit costs are described				
18	Currency and price data are recorded				
19	Details of currency of price adjustments for inflation or currency conversion are given				
20	Details of any model used are given				
21	The choice of model used and the key parameters on which it is based on justified				
**Analysis and interpretation of results**					

Confidential: For Review Only

22	Time horizon of costs and benefits is stated				
23	The discount rate(s) is stated				
24	The choice of discount rate(s) is justified				
25	An explanation is given if costs and benefits are not discounted				
26	Details of statistical tests and confidence intervals are given for stochastic data				
27	The approach to sensitivity analysis is given				
28	The choice of variables for sensitivity analysis is justified				
29	The ranges over which the variables are varied are justified				
30	Relevant alternatives are compared				
31	Incremental analysis is reported				
32	Major outcomes are presented in a disaggregated as well as aggregated form				
33	The answer to the study question is given				
34	Conclusions follow from the data reported				
35	Conclusions are accompanied by the appropriate caveats				

Confidential: For Review Only

Supplementary File 1

Title: Cost-Effectiveness of Neonatal Surgery for Congenital Anomalies in Low- and Middle-Income Countries: A Systematic Review Protocol

PRISMA-P (Preferred Reporting Items for Systematic review and Meta-Analysis Protocols) 2015 checklist: recommended items to address in a systematic review protocol

Section and Topic	Item No	Checklist Item	Section where each item is addressed in the protocol
**ADMINISTRATIVE INFORMATION**			
Title			
Identification	1a	Identify the report as a protocol of a systematic review	Title Page
Update	1b	If the protocol is for an update of a previous systematic review, identify as such	n/a
Registration	2	If registered, provide the name of the registry (such as PROSPERO) and registration number	Title Page
Authors:			
Contact	3a	Provide name, institutional affiliation, e-mail address of all protocol authors; provide physical mailing address of corresponding author	Title Page
Contributions	3b	Describe contributions of protocol authors and identify the guarantor of the review	Author's Contributions
Amendments	4	If the protocol represents an amendment of a previously completed or published protocol, identify as such and list changes; otherwise, state plan for documenting important protocol amendments	n/a
Support:			
Sources	5a	Indicate sources of financial or other support for the review	Funding Statement
Sponsor	5b	Provide name for the review funder and/or sponsor	Funding Statement
Role of sponsor or funder	5c	Describe roles of funder(s), sponsor(s), and/or institution(s), if any, in developing the protocol	Funding Statement
**INTRODUCTION**			
Rationale	6	Describe the rationale for the review in the context of what is already known	Introduction

Confidential: For Review Only

Objectives	7	Provide an explicit statement of the question(s) the review will address with reference to participants, interventions, comparators, and outcomes (PICO)	Aim and Objectives
**METHODS**			
Eligibility criteria	8	Specify the study characteristics (such as PICO, study design, setting, time frame) and report characteristics (such as years considered, language, publication status) to be used as criteria for eligibility for the review	Search Strategy; Inclusion and Exclusion
Information sources	9	Describe all intended information sources (such as electronic databases, contact with study authors, trial registers or other grey literature sources) with planned dates of coverage	Search Strategy
Search strategy	10	Present draft of search strategy to be used for at least one electronic database, including planned limits, such that it could be repeated	Supplementary File 2
Study Records:			
Data management	11a	Describe the mechanism(s) that will be used to manage records and data throughout the review	Study screening and Data Extraction
Selection process	11b	State the process that will be used for selecting studies (such as two independent reviewers) through each phase of the review (that is, screening, eligibility and inclusion in meta-analysis)	Study screening
Data collection process	11c	Describe planned method of extracting data from reports (such as piloting forms, done independently, in duplicate), any processes for obtaining and confirming data from investigators	Data extraction
Data items	12	List and define all variables for which data will be sought (such as PICO items, funding sources), any pre-planned data assumptions and simplifications	Data extraction
Outcomes and prioritization	13	List and define all outcomes for which data will be sought, including prioritization of main and additional outcomes, with rationale	Data extraction
Risk of bias in individual studies	14	Describe anticipated methods for assessing risk of bias of individual studies, including whether this will be done at the outcome or study level, or both; state how this information will be used in data synthesis	Methodological quality
Data synthesis	15a	Describe criteria under which study data will be quantitatively synthesized	Data synthesis
	15b	If data are appropriate for quantitative synthesis, describe planned summary measures, methods of handling data and methods of combining data from studies, including any planned exploration of consistency (such as I2, Kendall's τ)	Data synthesis

Confidential: For Review Only

	15c	Describe any proposed additional analyses (such as sensitivity or subgroup analyses, meta-regression)	n/a
	15d	If quantitative synthesis is not appropriate, describe the type of summary planned	n/a
Meta-bias(es)	16	Specify any planned assessment of meta-bias(es) (such as publication bias across studies, selective reporting within studies)	Data synthesis
Confidence in cumulative evidence	17	Describe how the strength of the body of evidence will be assessed (such as GRADE)	Data synthesis

Confidential: For Review Only

Supplementary File 2

Cost-Effectiveness of Neonatal Surgery for Congenital Anomalies in Low- and Middle-Income Countries: A Systematic Review Protocol

Database: Ovid MEDLINE(R) and Epub Ahead of Print, In-Process & Other Non-Indexed Citations, Daily and Versions(R) (from 1946 to March 2020)

Search strategy:

Search No	Search Terms	Results
1	exp Congenital Abnormalities/ or congenital anomal*.mp.	594246
2	exp Congenital Abnormalities/ or Congenital Abnormal*.mp.	589129
3	Congenital Malformation*.mp.	594677
4	Abdominal Wall Defect*.mp.	2180
5	Aganglionosis.mp.	1259
6	Anal Atresia.mp. or exp Anus, Imperforate/	2890
7	Imperforate Anus.mp. or exp Anus, Imperforate/	3085
8	Anorectal Malformation*.mp.	2144
9	Anorectal stenosis.mp.	71
10	Apple peel syndrome.mp.	19
11	Biliary atresia.mp. or exp Biliary Atresia/	4864
12	Birth Defect*.mp.	10209
13	Bladder Exstrophy.mp.	2565
14	Branchial Tag.mp.	0
15	Branchial Vestige.mp.	0
16	exp Bronchopulmonary Sequestration/ or Bronchopulmonary sequestration*.mp.	2103
17	exp Bronchogenic Cyst/ or Bronchogenic Cyst*.mp.	1896
18	Cervicoaural Fistula.mp.	3
19	exp Choledochal Cyst/ or Choledochal Cyst*.mp.	2972
20	Cleft Lip.mp.	18181
21	Cleft Palate.mp.	24115
22	Clubfoot.mp.	4312
23	Colonic Atresia.mp.	157
24	exp "Cystic Adenomatoid Malformation of Lung, Congenital"/ or Congenital Cystic adenomatoid malformation of lung*.mp.	938
25	exp Hernias, Diaphragmatic, Congenital/ or congenital diaphragmatic hernia*.mp.	6002
26	exp Heart Defects, Congenital/ or Congenital Heart Defect*.mp.	151926
27	Congenital Hydronephrosis.mp.	358
28	Conjoined Twins.mp. or exp Twins, Conjoined/	2100

Confidential: For Review Only

29	Cryptorchidism.mp.	10167
30	exp Diaphragmatic Eventration/ or Diaphragmatic Eventration*.mp.	985
31	Down Syndrome.mp.	28239
32	Duodenal atresia.mp.	896
33	Duodenal Obstruction.mp.	3938
34	Duodenal Web.mp.	106
35	Epispadias.mp.	1134
36	Exomphalos.mp. or exp Hernia, Umbilical/	3895
37	Fetal Malformation*.mp.	2027
38	Gastroschisis.mp. or exp GASTROSCHISIS/	2485
39	Hirschsprung disease.mp. or exp Hirschsprung Disease/	4885
40	Hydrocele.mp.	3117
41	Hypospadias.mp.	7034
42	Ileal Atresia.mp.	267
43	Imperforate Anus.mp. or exp Anus, Imperforate/	3085
44	Imperforate Hymen.mp.	304
45	Indeterminate Sex.mp.	2655
46	Intestinal Atresia.mp. or exp Intestinal Atresia/	24
47	Jejunal Atresia.mp.	276
48	Jejuno-Ileal Atresia.mp.	43
49	Malrotation.mp.	2596
50	exp Maxillofacial Abnormalities/ or Maxillofacial Abnormalit*.mp.	29759
51	exp Megacolon/ or Ganglionic Megacolon.mp.	7357
52	exp Mouth Abnormalities/ or Mouth Abnormalit*.mp.	25868
53	exp Neural Tube Defects/ or Neural Tube Defect*.mp.	30527
54	Esophageal Atresia.mp. or exp Esophageal Atresia/ or ?esophageal Atresia.mp.	4429
55	Omphalocele.mp. or exp Hernia, Umbilical/	4443
56	Orofacial Cleft*.mp.	1404
57	***Pectus Excavatum.mp.***	***2076***
58	Pes Cavus.mp.	614
59	Phimosis.mp.	1399
60	Polycystic Kidney Disease.mp.	7779
61	Polydactyly.mp.	3493
62	Preauricular Sinus.mp.	92
63	Rectosigmoid aganglionosis.mp.	19
64	Redundant Neck Fold.mp.	0
65	Renal Anomal*.mp.	1286
66	Spina Bifida.mp.	8686
67	Syndactyly.mp.	3594
68	Tongue Tie.mp.	259
69	exp Tracheoesophageal Fistula/ or Tracheo-?esophageal fistula*.mp.	3850

Confidential: For Review Only

70	Umbilical Hernia.mp. or exp Hernia, Umbilical/	4428
71	Undescended Testicle.mp.	267
72	exp Urogenital Abnormalities/ or Genito-urinary Anomalies.mp.	60563
73	Volvulus.mp. or exp Intestinal Volvulus/	9513
74	Webbed Neck.mp.	210
75	LMICs.mp.	3347
76	(low- and middle-income countr*).mp.	16149
77	exp Developing Countries/ or Developing countr*.mp.	124234
78	Low-resource setting*.mp.	3854
79	Underdeveloped Countries.mp.	901
80	low-income countries.mp.	5873
81	middle-income countries.mp.	16375
82	limited resource setting*.mp.	371
83	Africa South of the Sahara.mp. or exp "Africa South of the Sahara"/	204678
84	Sub-Saharan Africa.mp. or exp "Africa South of the Sahara"/	212529
85	Less Resourced communities.mp.	1
86	Afghanistan.mp. or exp AFGHANISTAN/	6757
87	Albania.mp. or exp ALBANIA/	1318
88	Algeria.mp. or exp ALGERIA/	4112
89	American Samoa.mp.	389
90	Angola.mp. or exp ANGOLA/	1581
91	Argentina.mp. or exp ARGENTiNA/	22048
92	Armenia.mp. or exp ARMENIA/	1844
93	Azerbaijan.mp. or exp AZERBAIJAN/	1942
94	Bangladesh.mp. or exp BANGLADESH/	15072
95	Belarus.mp. or exp "Republic of Belarus"/	2675
96	Belize.mp. or exp BELIZE/	956
97	Benin.mp. or exp BENIN/	3812
98	exp BHUTAN/ or Bhutan.mp.	788
99	Bolivia.mp. or exp BOLIVIA/	3994
100	(Bosnia and Herzegovina).mp.	2766
101	Botswana.mp. or exp BOTSWANA/	2616
102	Brazil.mp. or exp BRAZIL/	117532
103	Bulgaria.mp. or exp BULGARIA/	7995
104	Burkina Faso.mp. or exp Burkina Faso/	4491
105	Burundi.mp. or exp BURUNDI/	970
106	Cabo Verde.mp. or exp Cabo Verde/	259
107	Cambodia.mp. or exp CAMBODIA/	4712
108	Cameroon.mp. or exp CAMEROON/	7750
109	Central African Republic.mp. or exp Central African Republic/	1197
110	Chad.mp. or exp CHAD/	1282
111	China.mp. or exp CHINA/	254780

Confidential: For Review Only

112	Colombia.mp. or exp COLOMBIA/	14784
113	Comoros.mp. or exp COMOROS/	497
114	Democratic Republic of the Congo.mp. or exp "Democratic Republic of the Congo"/	5183
115	exp "Democratic Republic of the Congo"/ or DRC.mp.	5435
116	Republic of the Congo.mp. or exp Congo/	7009
117	Costa Rica.mp. or exp Costa Rica/	5628
118	Cote d'Ivoire.mp. or exp Cote d'Ivoire/	3878
119	Ivory Coast.mp. or exp Cote d'Ivoire/	3689
120	Cuba.mp. or exp CUBA/	6785
121	Djibouti.mp. or exp DJIBOUTI/	416
122	Dominica.mp. or exp DOMINICA/	488
123	Dominican Republic.mp. or exp Dominican Republic/	2397
124	Ecuador.mp. or exp ECUADOR/	5611
125	exp EGYPT/ or Egypt.mp.	21049
126	El Salvador.mp. or exp El Salvador/	1442
127	Equatorial Guinea.mp. or exp Equatorial Guinea/	473
128	Eritrea.mp. or exp ERITREA/	613
129	Eswatini.mp.	608
130	Ethiopia.mp. or exp ETHIOPIA/	17135
131	Fiji.mp. or exp FIJI/	1903
132	Gabon.mp. or exp GABON/	2091
133	exp GAMBIA/ or Gambia.mp.	3145
134	exp "GEORGIA (REPUBLIC)"/ or exp GEORGIA/ or Georgia.mp.	17132
135	Ghana.mp. or exp GHANA/	11476
136	Grenada.mp. or exp GRENADA/	343
137	Guatemala.mp. or exp GUATEMALA/	4348
138	Guinea.mp. or exp GUINEA-BISSAU/ or exp EQUATORIAL GUINEA/ or exp GUINEA/ or exp PAPUA NEW	162087
139	Guyana.mp. or exp GUYANA/	1244
140	Haiti.mp. or exp HAITI/	4089
141	Honduras.mp. or exp HONDURAS/	1970
142	India.mp. or exp INDIA/	144259
143	Indonesia.mp. or exp INDONESIA/	15689
144	exp Iran/ or Islamic Republic of Iran.mp.	25873
145	exp IRAQ/ or Iraq.mp.	10026
146	Jamaica.mp. or exp JAMAICA/	4642
147	Jordan.mp. or exp JORDAN/	7073
148	Kazakhstan.mp. or exp KAZAKHSTAN/	3546
149	Kenya.mp. or exp KENYA/	21398
150	Kiribati.mp. or exp Micronesia/	2071

Confidential: For Review Only

151	Democratic People's Republic of Korea.mp. or exp "Democratic People's Republic of Korea"/	286
152	Kosovo.mp. or exp KOSOVO/	936
153	Kyrgyz Republic.mp. or exp Kyrgyzstan/	1295
154	exp Laos/ or Lao PDR.mp.	2157
155	Laos.mp. or exp LAOS/	2831
156	Lebanon.mp. or exp LEBANON/	5916
157	Lesotho.mp. or exp LESOTHO/	740
158	Liberia.mp. or exp LIBERIA/	1896
159	Libya.mp. or exp LIBYA/	1681
160	Madagascar.mp. or exp MADAGASCAR/	5284
161	Malawi.mp. or exp MALAWI/	7525
162	Malaysia.mp. or exp MALAYSIA/	21032
163	Maldives.mp. or exp Indian Ocean Islands/	11762
164	Mali.mp. or exp MALI/	3983
165	Marshall Islands.mp. or exp Micronesia/	2087
166	Mauritania.mp. or exp MAURITANIA/	704
167	Mauritius.mp. or exp MAURITIUS/	1068
168	exp MEXICO/ or Mexico.mp.	59178
169	Micronesia.mp. or exp MICRONESIA/	2382
170	Moldova.mp. or exp MOLDOVA/	1005
171	Mongolia.mp. or exp MONGOLIA/	4524
172	Montenegro.mp. or exp MONTENEGRO/	851
173	Morocco.mp. or exp MOROCCO/	7733
174	Mozambique.mp. or exp MOZAMBIQUE/	3798
175	Myanmar.mp. or exp MYANMAR/	4000
176	Namibia.mp. or exp NAMIBIA	1764
177	Nauru.mp. or exp Micronesia/	2034
178	Nepal.mp. or exp NEPAL/	11112
179	Nicaragua.mp. or exp NICARAGUA/	2155
180	exp NIGER/ or Niger.mp.	13543
181	Nigeria.mp. or exp NIGERIA/	36528
182	North Macedonia.mp.	576
183	Pakistan.mp. or exp PAKISTAN/	23852
184	Papua New Guinea.mp. or exp Papua New Guinea/	5290
185	Paraguay.mp. or exp PARAGUAY/	1643
186	Peru.mp. or exp PERU/	12948
187	Philippines.mp. or exp PHILIPPINES/	11797
188	Romania.mp. or exp ROMANIA/	12465
189	exp Russia/ or Russian Federation.mp.	53823
190	Rwanda.mp. or exp RWANDA/	3387

Confidential: For Review Only

191	exp AMERICAN SAMOA/ or Samoa.mp. or exp SAMOA/ or exp "INDEPENDENT STATE OF SAMOA"/	1132
192	(Sao Tome and Principe).mp.	159
193	Senegal.mp. or exp SENEGAL/	7616
194	Serbia.mp. or exp SERBIA/	5306
195	Sierra Leone.mp. or exp Sierra Leone/	2429
196	Solomon Islands.mp. or exp Melanesia/	6645
197	Somalia.mp. or exp SOMALIA/	2203
198	South Africa.mp. or exp South Africa/	52729
199	South Sudan.mp. or exp South Sudan/	519
200	Sri Lanka.mp. or exp Sri Lanka/	8301
201	Saint Lucia.mp. or exp Saint Lucia/	124
202	(Saint Vincent and the Grenadines).mp.	63
203	exp SUDAN/ or Sudan.mp.	9256
204	Suriname.mp. or exp SURINAME/	1195
205	Swaziland.mp. or exp SWAZILAND/	920
206	Syrian Arab Republic.mp.	84
207	Syria.mp. or exp SYRIA/	2972
208	Tajikistan.mp. or exp TAJIKISTAN/	1014
209	Tanzania.mp. or exp TANZANIA/	14837
210	Thailand.mp. or exp THAILAND/	35644
211	exp Timor-Leste/ or Timor-Leste.mp.	397
212	East Timor.mp.	205
213	Togo.mp. or exp TOGO/	1693
214	Tonga.mp. or exp TONGA/	496
215	Tunisia.mp. or exp TUNISIA/	10115764
216	exp TURKEY/ or Turkey.mp.	52166
217	Turkmenistan.mp. or exp TURKMENISTAN/	764
218	Tuvalu.mp. or exp Micronesia/	2004
219	Uganda.mp. or exp UGANDA/	16218
220	Ukraine.mp. or exp UKRAINE/	17463
221	Uzbekistan.mp. or exp UZBEKISTAN/	2255
222	Vanuatu.mp. or exp VANUATU/	696
223	Venezuela.mp. or exp VENEZUELA/	7038
224	exp VIETNAM/ or Vietnam.mp.	18084
225	(West Bank and Gaza).mp.	221
226	Republic of Yemen.mp. or exp Yemen/	1377
227	Zambia.mp. or exp ZAMBIA/	6141
228	Zimbabwe.mp. or exp ZIMBABWE/	7550
229	Cost Effectiveness.mp.	59049
230	Cost Benefit Analys*.mp.	82018
231	Cost of Illness.mp.	27515

Confidential: For Review Only

232	Cost Effective Surgical Intervention*.mp.	162087
233	Health Care Cost*.mp.	49805
234	Disability Adjusted Life Year*.mp.	3263
235	DALY*.mp.	3036
236	Quality Adjusted Life Year*.mp.	17761
237	QALY*.mp.	10367
238	Health Adjusted Life Year*.mp.	45
239	HALY*.mp.	604
240	1 or 2 or 3 or 4 or 5 or 6 or 7 or 8 or 9 or 10 or 11 or 12 or 13 or 14 or 15 or 16 or 17 or 18 or 19 or 20 or 21 or 22 or 23 or 24 or 25 or 26 or 27 or 28 or 29 or 30 or 31 or 32 or 33 or 34 or 35 or 36 or 37 or 38 or 39 or 40 or 41 or 42 or 43 or 44 or 45 or 46 or 47 or 48 or 49 or 50 or 51 or 52 or 53 or 54 or 55 or 56 or 57 or 58 or 59 or 60 or 61 or 62 or 63 or 64 or 65 or 66 or 67 or 68 or 69 or 70 or 71 or 72 or 73 or 74	666909
241	75 or 76 or 77 or 78 or 79 or 80 or 81 or 82 or 83 or 84 or 85 or 86 or 87 or 88 or 89 or 90 or 91 or 92 or 93 or 94 or 95 or 96 or 97 or 98 or 99 or 100 or 101 or 102 or 103 or 104 or 105 or 106 or 107 or 108 or 109 or 110 or 111 or 112 or 113 or 114 or 115 or 116 or 117 or 118 or 119 or 120 or 121 or 122 or 123 or 124 or 125 or 126 or 127 or 128 or 129 or 130 or 131 or 132 or 133 or 134 or 135 or 136 or 137 or 138 or 139 or 140 or 141 or 142 or 143 or 144 or 145 or 146 or 147 or 148 or 149 or 150 or 151 or 152 or 153 or 154 or 155 or 156 or 157 or 158 or 159 or 160 or 161 or 162 or 163 or 164 or 165 or 166 or 167 or 168 or 169 or 170 or 171 or 172 or 173 or 174 or 175 or 176 or 177 or 178 or 179 or 180 or 181 or 182 or 183 or 184 or 185 or 186 or 187 or 188 or 189 or 190 or 191 or 192 or 193 or 194 or 195 or 196 or 197 or 198 or 199 or 200 or 201 or 202 or 203 or 204 or 205 or 206 or 207 or 208 or 209 or 210 or 211 or 212 or 213 or 214 or 215 or 216 or 217 or 218 or 219 or 220 or 221 or 222 or 223 or 224 or 225 or 226 or 227 or 228	1548976
242	229 or 230 or 231 or 232 or 233 or 234 or 235 or 236 or 237 or 238 or 239	337705
243	240 and 241 and 242	992

Confidential: For Review Only

BMJ Paediatrics Open

Cost-Effectiveness of Neonatal Surgery for Congenital Anomalies in Low- and Middle-Income Countries: A Systematic Review Protocol

Journal:	*BMJ Paediatrics Open*
Manuscript ID	bmjpo-2020-000755.R1
Article Type:	Protocol
Date Submitted by the Author:	21-Jul-2020

Complete List of Authors:	Kim, Na Eun; Boston Medical Center; King's College London<br>Vervoot, Dominique; Johns Hopkins Bloomberg School of Public Health<br>Hammouri, Ahmad; Al Quds University<br>Riboni, Cristiana; University of Pavia<br>Salem, Hosni; Cairo University<br>Grimes, Caris; King's College London; Medway NHS Foundation Trust, Department of Surgery<br>Wright, Naomi; King's Centre for Global Health and Health Partnerships

Keywords:	Neonatology

SCHOLARONE™
Manuscripts

BMJ

I, the Submitting Author has the right to grant and does grant on behalf of all authors of the Work (as defined in the below author licence), an exclusive licence and/or a non-exclusive licence for contributions from authors who are: i) UK Crown employees; ii) where BMJ has agreed a CC-BY licence shall apply, and/or iii) in accordance with the terms applicable for US Federal Government officers or employees acting as part of their official duties; on a worldwide, perpetual, irrevocable, royalty-free basis to BMJ Publishing Group Ltd ("BMJ") its licensees and where the relevant Journal is co-owned by BMJ to the co-owners of the Journal, to publish the Work in this journal and any other BMJ products and to exploit all rights, as set out in our licence.

The Submitting Author accepts and understands that any supply made under these terms is made by BMJ to the Submitting Author unless you are acting as an employee on behalf of your employer or a postgraduate student of an affiliated institution which is paying any applicable article publishing charge ("APC") for Open Access articles. Where the Submitting Author wishes to make the Work available on an Open Access basis (and intends to pay the relevant APC), the terms of reuse of such Open Access shall be governed by a Creative Commons licence – details of these licences and which Creative Commons licence will apply to this Work are set out in our licence referred to above.

Other than as permitted in any relevant BMJ Author's Self Archiving Policies, I confirm this Work has not been accepted for publication elsewhere, is not being considered for publication elsewhere and does not duplicate material already published. I confirm all authors consent to publication of this Work and authorise the granting of this licence.

Confidential: For Review Only

Cost-Effectiveness of Neonatal Surgery for Congenital Anomalies in Low- and Middle-Income Countries: A Systematic Review Protocol

Authors:
Na Eun Kim, MD
Boston Medical Center
88 East Newton St C515, Boston, Massachusetts, United States
Naeun88@gmail.com
(Corresponding author)

Dominique Vervoort, MD
Johns Hopkins Bloomberg School of Public Health
615 North Wolfe Street, 21205 Baltimore, Maryland, United States
vervoortdominique@hotmail.com

Ahmad Hammouri, MD
Al-Quds University Medical School
Al-Salam Street, Hebron, West-Bank, Palestine
ahmadalhammouri94@gmail.com

Cristiana Riboni
University of Pavia
C.so Strada Nuova, 65 - Pavia, Italy
cristiana.riboni01@universitadipavia.it

Hosni Salem, MD
Faculty of Medicine, Cairo University
Cairo, Egypt

Caris E Grimes
King's Centre for Global Health and Health Partnerships,
School of Population Health and Environmental Sciences,
Faculty of Life Sciences and Medicine, King's College London, London, UK.
and Department of Surgery, Medway NHS Foundation Trust, Windmill Road, Gillingham, Kent ME7 5NY. UK
Caris.grimes@kcl.ac.uk

Naomi Wright
King's Centre for Global Health and Health Partnerships,
School of Population Health and Environmental Sciences,
Faculty of Life Sciences and Medicine, King's College London, London, UK.
naomiwright@doctors.org.uk

Amendments:
Version 2

PROSPERO registration number
CRD 42020172971

Keywords: Neonatal Surgery, Paediatric Surgery, Congenital Anomalies, Cost-effectiveness, LMICs

Confidential: For Review Only

Abstract:

Introduction

Congenital anomalies are the 5th leading cause of death in under 5-year olds globally (591,000 deaths reported, 2016). Over 95% of deaths occur in low- and middle-income countries (LMICs). It is estimated that two-thirds of the congenital anomaly health burden could be averted through surgical intervention and that such interventions can be cost-effective. This systematic review aims to evaluate current evidence regarding the cost-effectiveness of neonatal surgery for congenital anomalies in LMICs.

Methods and analysis

A systematic literature review will be conducted in PubMed, MEDLINE, Embase, Cochrane Library, Scielo, Google scholar, African Journals OnLine, and Regional WHO's African Index Medicus databases for articles on the cost-effectiveness of neonatal surgery for congenital anomalies in LMICs. The following search strings will be used: 1. Congenital anomalies; 2. LMICs; 3. Cost-effectiveness of surgical interventions. Articles will be uploaded to Covidence software, duplicates removed and remaining articles screened by two independent reviewers. Cost information for interventions or procedures will be extracted by country and condition. Outcome measurements by reported unit and cost-effectiveness ratios will be extracted. Methodological quality of each article will be assessed using the Drummond checklist for economic evaluations. The Agency for Healthcare research and Quality's Effective Health Care Program (AHRQ) guidance will be followed to assess the grade of the studies.

Ethics and dissemination

No ethical approval is required for conducting the systematic review. There will be no direct collection of data from individuals. The finalized article will be published in a scientific journal for dissemination. The protocol has been registered with PROSPERO.

Conclusion

Congenital anomalies form a large component of the global health burden that is amenable to surgical intervention. This study will systematically review the current literature on the cost-effectiveness of neonatal surgery for congenital anomalies in LMICs.

Confidential: For Review Only

What is known about the subject:

- Congenital anomalies are a large component of the global health burden, recently rising to become the 5th leading cause of death in children under 5 years globally.
- Over 95% of deaths occur in low and middle income countries (LMICs).
- Surgical interventions have been shown to be cost-effective in reducing the burden of disease for some congenital anomalies.

What this study hopes to add:

- To provide an evidence based summary of the cost-effectiveness of neonatal surgery for congenital anomalies in LMICs to direct future interventions and investments.
- Identify specific congenital conditions that are the most cost-effective in LMICs.

Manuscript:

Introduction:

Congenital anomalies constitute a large global health burden, accounting for an estimated 591,000 deaths worldwide in 2016 [1] . The burden of disease from congenital anomalies falls most heavily on low- and middle-income countries (LMICs), where over 95% of the deaths from congenital anomalies occur [2, 3]. The burden of disease, traditionally expressed in disability-adjusted life years (DALYs), represents a health gap in a population due to early mortality and years of healthy life lost. As an example of this health gap, an estimated 140,154 DALYs are potential avertable by neonatal surgery in Uganda, however only 3.5% of the need is currently being met by the health system [4]. A study of four low income countries, Nepal, Rwanda, Sierra Leone and Uganda, showed 62% of the paediatric population had at least one unmet surgical need, approximating to 3.7 million children who need surgical care. These surgical conditions included congenital anomalies along with masses, wounds, burns and abdominal pain. This study reported congenital anomalies were less likely to be treated compared to wounds, burns and extremity conditions [5]. The higher burden in LMICs may also be attributed to a higher disease incidence related to a higher rate of micronutrient and macronutrient deficiencies, exposure to teratogens, intrauterine infections, and self-medication. In addition, elective pregnancy termination following prenatal diagnosis is less available in LMICs compared to high-income countries (HICs) [6].

Mortality rates for many congenital anomalies in LMICs are high. For example, gastroschisis mortality has been reported as 75-100% in many paediatric surgical centres across sub-Saharan Africa compared to less than 4% in HICs [6, 7, 8]. Disturbingly, published high mortality rates from congenital anomalies may even be an underestimate of the true burden of disease due to sizable hidden mortality. Children that do not reach health facilities and die at home or in transit are traditionally not accounted for [9, 10]. In 2014 the rate of facility-based delivery averaged 43% in developing countries and 48% in Sub-Saharan Africa [11]. Barriers to facility based delivery includes social and cultural factors, distance to the facility and cost of delivery [12]. EUROCAT national estimates show that infant mortality from congenital anomalies could be up to 29% higher than that reported by the World Health Organization (WHO) [13]. Furthermore, it is difficult to estimate the true burden of congenital anomalies in LMICs due to a lack of congenital anomaly registries and deficient research.

Traditionally, surgical care for neonates has been perceived as too complex and expensive to be prioritized or even implemented in LMICs, amidst competing public and global health priorities. However, a growing body of literature has arisen evaluating the potential of surgical interventions to reduce the burden of disease due to congenital anomalies in LMICs [14, 15, 16, 17]. 57% of DALYs caused by cleft lip and palate, congenital heart anomalies, and neural tube defects are potentially amenable to surgical care in LMICs [14]. Among gastroschisis patients in Uganda, up to 58.7 DALYs could be avertable per patient if appropriate surgical care was available [18]. Despite this high unmet need that can be prevented with surgical care, the burden of surgical disease in the paediatric population remains high in LMICs. Many surgical conditions are congenital, yet surgical care in LMICs is often delayed, worsening outcomes due to presenting with more advanced disease processes [15, 19]. In Kenya, only 13.5% of the required surgical interventions were performed for common congenital conditions requiring surgical care [17].

Surgical care has been found to be cost-effective in LMICs across various fields, including paediatric surgery [20, 21, 22]. Treating congenital anomalies such as cleft lip and palate, anorectal malformation, congenital diaphragmatic hernia, and congenital heart defects can have significant economic impact by adding to lifetime individual income and quality life lived [16, 23, 24, 25]. In addition, untreated conditions such as hydrocephalus in infants can exact a large economic burden, whereas treating them has been shown to have a favourable cost-benefit ratio [26]. Much has been added to the literature recently on the cost-effectiveness of surgery for paediatric conditions, including the systematic review by Saxton et al in 2016 on the cost-effectiveness of paediatric conditions in LMICs [22]. However, a systematic review of the cost-effectiveness studies for congenital anomalies in LMICs has not been conducted. Neonatal surgery is often deemed expensive due to the involvement of neonatal intensive care unit resources and specialists trained in neonates. Neonatal surgery uses a different set of resources and healthcare professionals compared to general paediatric surgery.

It is important to specifically analyse neonatal surgery independent of paediatric surgery and to fully understand the economic burden and the cost-effectiveness of surgical interventions for congenital anomalies to improve outcomes. This study aims to systematically review existing literature on the cost-effectiveness of neonatal surgery for the management of congenital anomalies in LMICs.

Aim: To conduct a systematic review that identifies and analyzes cost-effectiveness of neonatal surgical interventions for congenital anomalies in LMICs.

Objectives:

1. To systematically identify studies on cost-effectiveness of neonatal surgery for congenital anomalies in LMICs;
2. Evaluate the cost-effectiveness of neonatal surgery for congenital anomalies in LMICs;
3. To provide an evidence-based summary of cost-effectiveness of neonatal surgery for congenital anomalies in LMICs to direct future interventions and investments in neonatal surgery;
4. Identify specific congenital conditions that are most cost-effective in LMICs.
5. Critically appraise the quality of the studies included in the systematic review.

Methods and analysis

A systematic literature review will be conducted following the PRISMA-P guidelines (Supplementary file 1). Any amendments to the protocol will be reported in the publication of the results.

Inclusion/ Exclusion criteria:

Inclusion criteria:

Confidential: For Review Only

Conditions: Abdominal Wall Defect, Aganglionosis, Anal Atresia, Anorectal Malformation, Anorectal Stenosis, Apple Peel Syndrome, Biliary Atresia, Birth Defects, Bladder Exstrophy, Branchial Tag, Branchial Vestige, Bronchopulmonary Sequestration, Cervicoaural Fistula, Choledochal Cyst, Cleft Lip, Cleft Palate, Clubfoot, Colonic Atresia, Congenital Abnormalities, Congenital Anomalies, Congenital Cystic Adenomatoid Malformation of Lung, Congenital Diaphragmatic Hernia, Congenital Heart Defects, Congenital Hydronephrosis, Congenital Malformation, Conjoined Twins, Cryptorchidism, Diaphragmatic Eventration, Down Syndrome, Duodenal Atresia, Duodenal Obstruction, Duodenal Web, Epispadias, Exomphalos, Fetal Malformation, Gastroschisis, Hirschsprung's Disease, Hydrocele, Hypospadias, Ileal Atresia, Imperforate Anus, Imperforate Hymen, Indeterminate Sex, Intestinal Atresia, Jejunal Atresia, Jejuno-Ileal Atresia, Malrotation, Neural Tube Defects, Oesophageal Atresia, Omphalocele, Orofacial Clefts, Pectus Excavatum, Pes Cavus, Phimosis, Polycystic Kidney Disease, Polydactyly, Preauricular Sinus, Redundant Neck Fold, Spina Bifida, Syndactyly, Tongue Tie, Tracheo Oesophageal Fistula, Umbilical Hernia, Undescended Testicle, Volvulus, Webbed Neck

Age: Neonatal

Place: Low- and middle-income countries (as defined by World Bank 2019 Classification)

Intervention: Surgical or operative interventions performed in the antenatal setting and within the first 28 days of life.

Exclusion criteria:

Conditions: All conditions not listed in inclusion criteria

Age: Greater than 28 days of life

Place: High-income countries.

Intervention: Any procedure performed after the first 28 days of life will be excluded.

Search strategy:
A systematic literature search will be conducted using the following search strings: 1. Congenital anomalies; 2. LMICs; 3. Cost-effectiveness of surgical interventions (Table 1). The following databases will be used: PubMed, MEDLINE, EMBASE, Cochrane Library, Scielo, Google scholar, African Journals OnLine, and Regional WHO's African Index Medicus. The search will include English, French, Spanish, Italian, and Arabic articles and will be restricted to human studies. There will be no restrictions on publication date or study design.

Study design
Published, peer-reviewed journal articles will be included. Any study without explicit cost data or health outcomes data will be excluded. Case reports, editorials, letters to the editor, and literature reviews will be excluded. Abstracts without available full text will be excluded.

Methodological quality
The studies will be assessed for their methodological quality of economic evaluations using the Drummond 10-point checklist [27] (Table 2). The results will be summarized in a table format in the results section. In addition to the Drummond checklist, the Agency for Healthcare research and Quality's Effective Health Care Program (AHRQ) guidance will be followed to assess the grade of the studies. The domains included in this assessment are risk of bias, consistency, directness and precision. The strength of evidence will be categorized into high, moderate, low and insufficient groups [28].

Study screening
Articles will be uploaded to Covidence software (Melbourne, Australia), duplicate articles will be removed, and remaining articles will be screened by two independent reviewers. All potential eligible articles will be screened in full text for final selection by two independent reviewers. The reference lists of the included articles will be screened to identify further eligible studies. Conflicts between the two reviewers' assessments will be resolved by a consensus meeting of all authors.

Data extraction
The following data will be extracted: study type or design, study population, study period, country, year of publication, journal, author name(s), number of patients, patient demographics, type of condition, type of intervention, type of healthcare system, gestational age, weight, time from birth to presentation, costs incurred during treatment, outcome of intervention (mortality and morbidity which will be reported using the Clavien-Dindo scoring system) [29].

Main outcomes will be the cost of surgical interventions and health outcomes. The effectiveness method of DALYs, HALYs, and QALYs will be extracted. Reported incremental cost-effectiveness ratio and/or potential gains in life expectancy will be extracted. Studies without a comparison to a prior intervention and reporting only a cost effectiveness ratio will also be included in our study. Data reported for different countries or procedures will be extracted as separate results.

The WHO-CHOICE guidelines will be used to determine the interventions' cost effectiveness category. Cost effective intervention thresholds will be defined by the WHO based on the GDP per capita per DALY averted. Those that cost less than the GDP per capita per DALY averted will be categorized as very cost effective, interventions that cost one to three times the GDP per capita per DALY as cost effective and those that cost more than three times the GDP per capita per DALY will be determined as not cost effective [30].

Confidential: For Review Only

Data synthesis

The data will be organized into: author(s), the year of publication, intervention or procedure, country or site of intervention, gross domestic product, costs per outcome, outcome unit, and currency.

Cost data will be organized into cost to the provider and cost to the patient. The cost will be converted to USD, calculated by the currency year. Quantitative analysis will be undertaken by calculating the median values and interquartile range for the intervention or procedures. Meta-analysis will likely not be feasible due to the limited availability of data. If our search results in the appropriate data a meta-analysis will be conducted. Appropriate data is defined as two or more homogeneous studies comparing the cost and outcomes of a specific surgical procedure to another non-surgical intervention. The meta-analysis will be conducted in Stata.

The GRADE (Grading of Recommendations Assessment, Development, and Evaluation) system will be used to rate the quality of evidence and the strength of recommendations.

Patient and public involvement

Whilst we recognize the importance of public and patient involvement in research, on this occasion it has not been feasible to incorporate this into the design of the study due to difficulty engaging with patients and their families affected by congenital anomalies within LMICs in such a project. We will endeavour to ensure that a summary of the results of the study is provided in lay language and disseminated for public viewing.

Ethics and Dissemination

No ethical approval was required for conducting the systematic review. There was no direct collection of data from individuals. The finalized article will be published in a scientific journal for dissemination.

Limitations

The studies may have been conducted in different settings, such as government facilities, non-profit hospitals, and surgical mission trips, which may limit direct comparisons. Costs may also be reported that vary in the components included. In addition, the health outcome calculations will be different by study, which may or may not include age weighting or discounting. The search engines used may not identify all articles. Lastly, it is unlikely that the incremental cost-effectiveness ratio will be directly comparable among studies as the alternative comparison may be different for each study.

Discussion:

Congenital anomalies are reported to account for approximately 62.9 million or 2.4% of global DALYs in 2016 [31]. Published literature from LMICs commonly describe the burden of disease from congenital disease in terms of mortality instead of DALYs, emphasizing the difference in mortality rates compared to those from HICs. Reducing infant mortality due to congenital anomalies will help to meet the Sustainable Development Goals (SDG) aim to reduce preventable deaths in neonates to as low as 12 per 1,000 live births by 2030 [32]. Current literature has shown that surgical care for congenital anomalies can reduce the burden of disease in a cost-effective manner [16, 21, 23, 24, 33]. An estimated two-thirds of the burden of disease related to congenital anomalies can be averted through surgical care [34]. Despite this potential, the burden of surgical disease in this neonatal population remains high in LMICs.

There is a growing body of literature for cost effectiveness of surgical interventions for congenital anomalies but to our knowledge there has been no systematic analysis consolidating these studies. The most recent literature review by *Stolk et al* was completed in 2000 reporting two complete economic evaluations of cost-effectiveness in neonatal surgery for congenital anomalies which were not done in the context of LMICs [33, 35, 36]. This project will provide a crucial addition to the literature by providing a systematic review on current literature of cost effectiveness analysis of neonatal surgery for congenital anomalies in LMICs.

Supplementary files

Supplementary file 1: Prisma checklist
Supplementary file 2: Example Search Strategy

Authors' contributions

All authors contributed to the design of the systematic review and drafted the manuscript including its revisions. All authors gave final approval of the version to be published and agree to be accountable for all aspects of the work and ensure that questions related to the accuracy of the work are appropriately investigated and resolved.

Funding statement:

Naomi Wright receives funding from the Wellcome Trust to undertake a Clinical PhD in Global Health at King's Centre for Global Health and Health Partnerships, King's College London (Funder Reference: 203905/Z/16/Z).
No financial relationships

Competing interests:

There are no conflicts of interests.

Confidential: For Review Only

References

1. WHO 2016. Disease burden and mortality estimates. https://www.who.int/healthinfo/global_burden_disease/estimates/en/ (Accessed 6 March 2020).
2. Sitkin NA, Farmer DL. Congenital anomalies in the context of global surgery. Semin Pediatr Surg. 2016 Feb;25(1):15-8. doi: 10.1053/j.sempedsurg.2015.09.004. Epub 2015 Sep 16.
3. WHO 2012. Mortality Statistics. http:// apps. who. int/ healthinfo/ statistics/ mortality/who. htm (accessed 7 July 2019).
4. Badrinath R, Kakembo N, Kisa P, et al. Outcomes and unmet need for neonatal surgery in a resource-limited environment: estimates of global health disparities from Kampala, Uganda. J Pediatr Surg. 2014 Dec;49(12):1825-30. doi: 10.1016/j.jpedsurg.2014.09.031. Epub 2014 Oct 1.
5. Butler EK, Tran TM, Nagarajan N, et al. Epidemiology of pediatric surgical needs in low-income countries. *PLoS One*. 2017;12(3):e0170968. Published 2017 Mar 3. doi:10.1371/journal.pone.0170968
6. Sitkin NA, Ozgediz D, Donkor P, et al. Congenital anomalies in low- and middle income countries: the unborn child of global surgery. World J Surg 2015;39:36–40.
7. Wright NJ, Zani A, Ade-Ajayi N. Epidemiology, management and outcome of gastroschisis in Sub-Saharan Africa: Results of an international survey. *Afr J Paediatr Surg* 2015; **12**(1): 1-6.
8. Bradnock T, Marven S, Owen A, et al. Gastroschisis: one year outomes from national cohort study. *BMJ* 2011; **343**(d6749).
9. Modell B, Berry RJ, Boyle CA, et al. Global regional and national causes of child mortality. Lancet 2012;380:1556.
10. Smith ER, Concepcion TL, Shrime M, et al. Waiting Too Long: The Contribution of Delayed Surgical Access to Pediatric Disease Burden in Somaliland. *World J Surg*. 2020;44(3):656-664. doi:10.1007/s00268-019-05239-w
11. UNICEF: The State of the World's Children 2014 in Numbers: Every Child Counts. 2014. http://www.unicef.org/publications/index_71829.html (Accessed 16 July 2020).
12. Bohren MA, Hunter EC, Munthe-Kaas HM, Souza JP, Vogel JP, Gülmezoglu AM. Facilitators and barriers to facility-based delivery in low- and middle-income countries: a qualitative evidence synthesis. *Reprod Health*. 2014;11(1):71. Published 2014 Sep 19. doi:10.1186/1742-4755-11-71
13. Boyle B, Addor MC, Arriola L, et al. Estimating Global Burden of Disease due to congenital anomaly: an analysis of European data. *Arch Dis Child Fetal Neonatal Ed*. 2018;103(1):F22-F28. doi:10.1136/archdischild-2016-311845
14. Higashi, H., J. J. Barendregt, and T. Vos. 2013. "The Burden of Congenital Anomalies Amenable to Surgeries in Low-Income and Middle-Income Countries: A Modeled Analysis." The Lancet 381: S62.
15. Ozgediz, D., and D. Poenaru. 2012. "The Burden of Pediatric Surgical Conditions in Low and Middle Income Countries: A Call to Action." Journal of Pediatric Surgery 47 (12): 2305–11.
16. Corlew, D. 2010. "Estimation of Impact of Surgical Disease through Economic Modeling of Cleft Lip and Palate Care." World Journal of Surgery 34 (3): 391–96.
17. Wu V, Poenaru D. 2013. Burden of surgically correctable disabilities among children in the Dadaab refugee camp. World J Surg 37:1536–1543. doi:10.1007/s00268-012-1899-z
18. Ford K, Poenaru D, Moulot O, et al. Gastroschisis: Bellwether for neonatal surgery capacity in low resource settings? J Pediatr Surg. 2016 Aug;51(8):1262-7. doi: 10.1016/j.jpedsurg.2016.02.090. Epub 2016 Mar 12.
19. Poenaru, D., J. Pemberton, C. Frankfurter, et al. 2013. "Establishing Disability Weights for Congenital Pediatric Surgical Disease: A Multi-modal Study." The Lancet 381: S3115
20. Chao TE, Sharma K, Mandigo M, et al. Cost-effectiveness of surgery and its policy implications for global health: a systematic review and analysis. *Lancet Glob Health*. 2014;2(6):e334-e345. doi:10.1016/S2214-109X(14)70213-X
21. Grimes CE, Henry JA, Maraka J, et al. Cost-effectiveness of surgery in low- and middle-income countries: a systematic review. World J Surg. 2014 Jan;38(1):252-63. doi: 10.1007/s00268-013-2243-y.
22. Saxton AT, Poenaru D, Ozgediz D, et al. Economic Analysis of Children's Surgical Care in Low- and Middle-Income Countries: A Systematic Review and Analysis. *PLoS One*. 2016;11(10):e0165480. Published 2016 Oct 28. doi:10.1371/journal.pone.0165480
23. Poley MJ, Brouwer WB, Busschbach JJ, et al. Cost-effectiveness of neonatal surgery: first greeted with scepticism, now increasingly accepted. Pediatr Surg Int. 2008 Feb;24(2):119-27. Epub 2007 Nov 6.
24. Cardarelli M, Vaikunth S, Mills K, et al. Cost-effectiveness of Humanitarian Pediatric Cardiac Surgery Programs in Low and Middle Income Countries. JAMA Netw Open. 2018 Nov 2;1(7):e184707. doi: 10.1001/jamanetworkopen.2018.4707.
25. Poley MJ, Stolk EA, Langemeijer RA, Molenaar JC, Busschbach JJ. The cost-effectiveness of neonatal surgery and subsequent treatment for congenital anorectal malformations. *J Pediatr Surg*. 2001;36(10):1471-1478. doi:10.1053/jpsu.2001.27025
26. Warf, B. C., B. C. Alkire, S. Bhai, et al. 2011. "Costs and Benefits of Neurosurgical Intervention for Infant Hydrocephalus in Sub-Saharan Africa." Journal of Neurosurgical Pediatrics 8 (5): 509–21.
27. Drummond, M. F., Sculpher, Mark J, Claxton, Karl, et al. Methods for the Economic Evaluation of Health Care Programmes. 4th ed. Oxford: OUP Oxford, 2015. Web.
28. Owens DK, Lohr KN, Atkins D, et al. AHRQ series paper 5: grading the strength of a body of evidence when comparing medical interventions--agency for healthcare research and quality and the effective health-care program. *J Clin Epidemiol*. 2010;63(5):513-523. doi:10.1016/j.jclinepi.2009.03.009
29. Dindo D, Demartines N, Clavien PA. Classification of surgical complications: a new proposal with evaluation in a cohort of 6336 patients and results of a survey. Ann Surg. 2004;240(2):205–13.
30. Edejer et al 2003. WHO: Making choices in health. Guide to Cost-effectiveness analysis. ISBN 92 4 154601 8.
31. Global Health Estimates 2016: Disease burden by Cause, Age, Sex, by Country and by Region, 2000-2016. Geneva, World Health Organization
32. United Nations. Sustainable Development Goals. 2015 ht tps://sustainabledevelopment. un. org/ ? me nu= 1300 (accessed 26 Jan 2016).
33. Stolk EA, Post HA, Rutten FF, et al. Cost-effectiveness of neonatal surgery: a review. J Pediatr Surg. 2000 Apr;35(4):588-92.

For peer review only

34. Debas HT, Donkor P, Gawande A, et al. Disease control priorities, 3rd edn—volume 1: essential surgery. Washington, DC: World Bank, 2015.
35. Roberts TE. Economic evaluation and randomised controlled trial of extracorporeal membrane oxygenation: UK collaborative trial. the Extracorporeal Membrane Oxygenation Economics Working Group. BMJ. 1998 Oct 3;317(7163):911-5; discussion 915-6.
36. Hackam DJ, Superina RA, Pearl RH. Single-stage repair of Hirschsprung's disease: A comparison of 109 patients over 5 year. J Pediatr Surg 32:1028-1031, 1997

Tables:

Table 1: Search Strategy
The following search strings were used during the literature search for this systematic review (Table 1). Search string 1 encompasses congenital anomalies found in neonates that commonly require surgical intervention. Search string 2 includes all low- and middle- income countries. Search string 3 describes the cost-effectiveness of an intervention.

Search String 1	*Search String 2*	*Search String 3*
Congenital Abnormalities, Congenital Anomalies, Congenital Malformation, Abdominal Wall Defect, Aganglionosis, Anal Atresia, Anal Stenosis, Anorectal Malformation, Anorectal Stenosis, Apple Peel Syndrome, Biliary Atresia, Birth Defects, Bladder Exstrophy, Branchial Tag, Branchial Vestige, Bronchopulmonary Sequestration, Bronchogenic Cyst, Cervicoaural Fistula, Choledochal Cyst, Cleft Lip, Cleft Palate, Clubfoot, Colonic Atresia, Congenital Cystic Adenomatoid Malformation of Lung, Congenital Diaphragmatic Hernia, Congenital Heart Defects, Congenital Hydronephrosis, Conjoined Twins, Cryptorchidism, Diaphragmatic Eventration, Down Syndrome, Duodenal Atresia, Duodenal Obstruction, Duodenal Web, Epispadias, Exomphalos, Fetal Malformation, Gastroschisis, Hirschsprung's Disease, Hydrocele, Hypospadias, Ileal Atresia, Imperforate Anus, Imperforate Hymen, Indeterminate Sex, Intestinal Atresia, Jejunal Atresia, Jejuno-Ileal Atresia, Malrotation, Maxillofacial Abnormalities, Megacolon, Mouth Abnormalities, Neural Tube Defects, Oesophageal Atresia, Omphalocele, Orofacial Clefts, Pectus Excavatum, Pes Cavus, Phimosis, Polycystic Kidney Disease, Polydactyly, Preauricular Sinus, Rectosigmoid Aganglionosis, Redundant Neck Fold, Renal Anomalies, Spina Bifida, Syndactyly, Tongue Tie, Tracheo-Oesophageal Fistula, Umbilical Hernia, Undescended Testicle, Urogenital Abnormalities, Volvulus, Webbed Neck	LMICs, Low- and Middle-Income Countries, Developing Countries, Low Resource Settings, Underdeveloped Countries, Low-Income Countries, Middle-Income Countries, Limited Resource Settings, Africa South of the Sahara, Sub-Saharan Africa, Less Resourced Communities, Afghanistan, Albania, Algeria, American Samoa, Angola, Argentina, Armenia, Azerbaijan, Bangladesh, Belarus, Belize, Benin, Bhutan, Bolivia, Bosnia and Herzegovina, Botswana, Brazil, Bulgaria, Burkina Faso, Burundi, Cabo Verde, Cambodia, Cameroon, Central African Republic, Chad, China, Colombia, Comoros, Democratic Republic of the Congo, DRC, Republic of the Congo, Costa Rica, Cote d'Ivoire, Ivory Coast, Cuba, Djibouti, Dominica, Dominican Republic, Ecuador, Egypt, Egypt Arab Republic, El Salvador, Equatorial Guinea, Eritrea, Eswatini, Ethiopia, Fiji, Gabon, Gambia, Georgia, Ghana, Grenada, Guatemala, Guinea, Guinea-Bissau, Guyana, Haiti, Honduras, India, Indonesia, Islamic Republic of Iran, Iraq, Jamaica, Jordan, Kazakhstan, Kenya, Kiribati, Democratic People's Republic of Korea, Kosovo, Kyrgyz Republic, Lao PDR, Laos, Lebanon, Lesotho, Liberia, Libya, Madagascar, Malawi, Malaysia, Maldives, Mali, Marshall Islands, Mauritania, Mauritius, Mexico, Micronesia, Moldova, Mongolia, Montenegro, Morocco, Mozambique, Myanmar, Namibia, Nauru, Nepal, Nicaragua, Niger, Nigeria, North Macedonia, Pakistan, Papua New Guinea, Paraguay, Peru, Philippines, Romania, Russian Federation, Rwanda, Samoa, Sao Tome and Principe, Senegal,	Cost, Cost Effectiveness, Cost Benefit Analysis, Cost of Illness, Cost Effective Surgical Intervention, Health Care Cost, DALYs, Disability Adjusted Life Years, QALYs, Quality Adjusted Life Years, HALYs, Health Adjusted Life Years

Confidential: For Review Only

	Serbia, Sierra Leone, Solomon Islands, Somalia, South Africa, South Sudan, Sri Lanka, Saint Lucia, Saint Vincent and the Grenadines, Sudan, Suriname, Swaziland, Syrian Arab Republic, Tajikistan, Tanzania, Thailand, Timor-Leste, Togo, Tonga, Tunisia, Turkey, Turkmenistan, Tuvalu, Uganda, Ukraine, Uzbekistan, Vanuatu, Venezuela, Vietnam, West Bank and Gaza, Republic of Yemen, Zambia, Zimbabwe, Kabul, Porto-Novo, Hogbonou, Adjace, Cotonou, Kutonu, Ouagadougou, Ouaga, Bujumbura, Usumbura, Phnom Penh, Bangui, Bangi, N'Djamena, Ndjamena, Fort Lamy, Moroni, Kinshasa, Asmara, Asmera, Addis Ababa, Addis Abeba, Banjul, Bathurst, Conakry, Bissau, Port-au-Prince, Pyongyang, Monrovia, Antananarivo, Tananarive, Tana, Lilongwe, Bamako, Maputo, Lourenco Marques, Kathmandu, Niamey, Kigali, Freetown, Free-town, Mogadishu, Xamar, Hamar, Muqdisho, Maqadishu, Juba, Dodoma, Dar es Salaam, Lome, Kampala, Harare, Salisbury, Yerevan, Dhaka, Dacca, Thimphu, Thimbu, Sucre, Charcas, La Plata, Chuquisaca, La Paz, Praia, Yaounde, Jaunde, Brazzaville, Yamoussoukro, Cairo, Accra, Tegucigalpa, Tegus, New Delhi, Jakarta, Nairobi, South Tarawa, Tarawa Teinainano, Pristina, Prishtina, Bishkek, Pishpek, Frunze, Vientiane, Maseru, Nouakchott, Palikir, Chisinau, Kishinev, Rabat, Nay Pyi Taw, Naypyidaw, Nepranytau, Naypyitaw, Kyetpyay, Pyinmana, Kyatpyay, Pyinmana, Yangon, Rangoon, Managua, Abuja, Lagos, Islamabad, Port Moresby, Moresby, Pom Town, Manila, Apia, Dakar, Honiara, Jayawardenepura, Jayewardenepura, Khartoum, Mbabane, Embabane, Lobamba, Damascus, Dushanbe, Dyushambe, Stalinabad, Dili, Kyiv, Kiev, Tashkent, Toshkent, Port Vila, Hanoi, Ha Noi, Sana'a, Sanaa, Sana, Lusaka, Ulaanbaatar, Ulan-Bator, Luanda, Tbilisi, Amman	

Table 2: Drummond Checklist

	Item	Yes (Page number)	No	Not Clear	Not Appropriate
	**Study Design**				
1	The research question is stated				
2	The economic importance of the research question is stated				
3	The viewpoint(s) of the analysis are clearly stated and justified				
4	The rationale for choosing alternative programmes or interventions compared is stated				
5	The alternatives being compared are clearly described				
6	The form of economic evaluation used is stated				
7	The choice of form of economic evaluation is justified in relation to the questions addressed				
	**Data Collection**				
8	The source(s) of effectiveness estimates used are stated				
9	Details of the design and results of effectiveness study are given (if based on a single study)				
10	Details of the methods of synthesis or meta-analysis of estimates are given (if based on a synthesis of a number of effectiveness studies)				
11	The primary outcome measure(s) for the economic evaluation are clearly stated				
12	Methods to value benefits are stated				
13	Details of the subjects from whom valuations were obtained were given				
14	Productivity changes (if included) are reported separately				
15	The relevance of productivity changes to the study question is discussed				
16	Quantities of resource use are reported separately from their unit costs				
17	Methods for the estimation of quantities and unit costs are described				
18	Currency and price data are recorded				
19	Details of currency of price adjustments for inflation or currency conversion are given				
20	Details of any model used are given				
21	The choice of model used and the key parameters on which it is based on justified				
	**Analysis and interpretation of results**				
22	Time horizon of costs and benefits is stated				
23	The discount rate(s) is stated				
24	The choice of discount rate(s) is justified				
25	An explanation is given if costs and benefits are not discounted				
26	Details of statistical tests and confidence intervals are given for stochastic data				
27	The approach to sensitivity analysis is given				
28	The choice of variables for sensitivity analysis is justified				
29	The ranges over which the variables are varied are justified				
30	Relevant alternatives are compared				
31	Incremental analysis is reported				
32	Major outcomes are presented in a disaggregated as well as aggregated form				
33	The answer to the study question is given				
34	Conclusions follow from the data reported				
35	Conclusions are accompanied by the appropriate caveats				

Confidential: For Review Only

Supplementary File 1

Title: Cost-Effectiveness of Neonatal Surgery for Congenital Anomalies in Low- and Middle-Income Countries: A Systematic Review Protocol

PRISMA-P (Preferred Reporting Items for Systematic review and Meta-Analysis Protocols) 2015 checklist: recommended items to address in a systematic review protocol

Confidential: For Review Only

Section and Topic	Item No	Checklist Item	Section where each item is addressed in the protocol
**ADMINISTRATIVE INFORMATION**			
Title			
Identification	1a	Identify the report as a protocol of a systematic review	Title Page
Update	1b	If the protocol is for an update of a previous systematic review, identify as such	n/a
Registration	2	If registered, provide the name of the registry (such as PROSPERO) and registration number	Title Page
Authors:			
Contact	3a	Provide name, institutional affiliation, e-mail address of all protocol authors; provide physical mailing address of corresponding author	Title Page
Contributions	3b	Describe contributions of protocol authors and identify the guarantor of the review	Author’s Contributions
Amendments	4	If the protocol represents an amendment of a previously completed or published protocol, identify as such and list changes; otherwise, state plan for documenting important protocol amendments	n/a
Support:			
Sources	5a	Indicate sources of financial or other support for the review	Funding Statement
Sponsor	5b	Provide name for the review funder and/or sponsor	Funding Statement
Role of sponsor or funder	5c	Describe roles of funder(s), sponsor(s), and/or institution(s), if any, in developing the protocol	Funding Statement
**INTRODUCTION**			
Rationale	6	Describe the rationale for the review in the context of what is already known	Introduction

Objectives	7	Provide an explicit statement of the question(s) the review will address with reference to participants, interventions, comparators, and outcomes (PICO)	Aim and Objectives
**METHODS**			
Eligibility criteria	8	Specify the study characteristics (such as PICO, study design, setting, time frame) and report characteristics (such as years considered, language, publication status) to be used as criteria for eligibility for the review	Search Strategy; Inclusion and Exclusion
Information sources	9	Describe all intended information sources (such as electronic databases, contact with study authors, trial registers or other grey literature sources) with planned dates of coverage	Search Strategy
Search strategy	10	Present draft of search strategy to be used for at least one electronic database, including planned limits, such that it could be repeated	Supplementary File 2
Study Records:			
Data management	11a	Describe the mechanism(s) that will be used to manage records and data throughout the review	Study screening and Data Extraction
Selection process	11b	State the process that will be used for selecting studies (such as two independent reviewers) through each phase of the review (that is, screening, eligibility and inclusion in meta-analysis)	Study screening
Data collection process	11c	Describe planned method of extracting data from reports (such as piloting forms, done independently, in duplicate), any processes for obtaining and confirming data from investigators	Data extraction
Data items	12	List and define all variables for which data will be sought (such as PICO items, funding sources), any pre-planned data assumptions and simplifications	Data extraction
Outcomes and prioritization	13	List and define all outcomes for which data will be sought, including prioritization of main and additional outcomes, with rationale	Data extraction
Risk of bias in individual studies	14	Describe anticipated methods for assessing risk of bias of individual studies, including whether this will be done at the outcome or study level, or both; state how this information will be used in data synthesis	Methodological quality
Data synthesis	15a	Describe criteria under which study data will be quantitatively synthesized	Data synthesis
	15b	If data are appropriate for quantitative synthesis, describe planned summary measures, methods of handling data and methods of combining data from studies, including any planned exploration of consistency (such as I2, Kendall's τ)	Data synthesis

Confidential: For Review Only

	15c	Describe any proposed additional analyses (such as sensitivity or subgroup analyses, meta-regression)	n/a
	15d	If quantitative synthesis is not appropriate, describe the type of summary planned	n/a
Meta-bias(es)	16	Specify any planned assessment of meta-bias(es) (such as publication bias across studies, selective reporting within studies)	Data synthesis
Confidence in cumulative evidence	17	Describe how the strength of the body of evidence will be assessed (such as GRADE)	Data synthesis

Confidential: For Review Only

Supplementary File 2b

Cost-Effectiveness of Neonatal Surgery for Congenital Anomalies in Low- and Middle-Income Countries: A Systematic Review Protocol

Database: Ovid MEDLINE(R) and Epub Ahead of Print, In-Process & Other Non-Indexed Citations, Daily and Versions(R) (from 1946 to March 2020)

Search strategy:

Search Number	Search term	Results
1	cost benefit analys*.mp.	83470
2	cost effectiveness.mp.	60533
3	cost of illness.mp.	28218
4	DALY*.mp.	3158
5	Disability Adjusted Life Year*.mp.	3402
6	HALY*.mp.	618
7	Health Adjusted Life Year*.mp.	49
8	Health Care Cost*.mp.	50825
9	QALY*.mp.	10711
10	Quality Adjusted Life Year*.mp.	18335
**11**	**1 or 2 or 3 or 4 or 5 or 6 or 7 or 8 or 9 or 10**	**179488**
12	exp Congenital Abnormalities/ or congenital anomal*.mp.	600611
13	exp Congenital Abnormalities/ or Congenital Abnormal*.mp.	595316
14	Congenital Malformation*.mp.	15162
15	Abdominal Wall Defect*.mp.	2217
16	Aganglionosis.mp.	1273
17	Anal Atresia.mp. or exp Anus, Imperforate/	2904
18	Imperforate Anus.mp. or exp Anus, Imperforate/	3103
19	Anorectal Malformation*.mp.	2208
20	Anorectal stenosis.mp.	71
21	Apple peel syndrome.mp.	18
22	Biliary atresia.mp. or exp Biliary Atresia/	4957
23	Birth Defect*.mp.	10388
24	Bladder Exstrophy.mp.	2590
25	Branchial Tag.mp.	0
26	Branchial Vestige.mp.	0
27	exp Bronchopulmonary Sequestration/ or Bronchopulmonary sequestration*.mp.	2122
28	exp Bronchogenic Cyst/ or Bronchogenic Cyst*.mp.	1924

Confidential: For Review Only

29	Cervicoaural Fistula.mp.	3
30	exp Choledochal Cyst/ or Choledochal Cyst*.mp.	3001
31	Cleft Lip.mp.	18452
32	Cleft Palate.mp.	24399
33	Clubfoot.mp.	4358
34	Colonic Atresia.mp.	160
35	exp "Cystic Adenomatoid Malformation of Lung, Congenital"/ or Congenital Cystic adenomatoid malformation of lung*.mp.	948
36	exp Hernias, Diaphragmatic, Congenital/ or congenital diaphragmatic hernia*.mp.	6097
37	exp Heart Defects, Congenital/ or Congenital Heart Defect*.mp.	153690
38	Congenital Hydronephrosis.mp.	363
39	Conjoined Twins.mp. or exp Twins, Conjoined/	2111
40	Cryptorchidism.mp.	10253
41	exp Diaphragmatic Eventration/ or Diaphragmatic Eventration*.mp.	991
42	Down Syndrome.mp.	28528
43	Duodenal atresia.mp.	903
44	Duodenal Obstruction.mp.	3962
45	Duodenal Web.mp.	107
46	Epispadias.mp.	1149
47	Exomphalos.mp. or exp Hernia, Umbilical/	3920
48	Fetal Malformation*.mp.	2053
49	Gastroschisis.mp. or exp GASTROSCHISIS/	2516
50	Hirschsprung disease.mp. or exp Hirschsprung Disease/	4949
51	Hydrocele.mp.	3159
52	Hypospadias.mp.	7140
53	Ileal Atresia.mp.	271
54	Imperforate Anus.mp. or exp Anus, Imperforate/	3103
55	Imperforate Hymen.mp.	307
56	Intestinal Atresia.mp. or exp Intestinal Atresia/	2680
57	Indeterminate Sex.mp.	24
58	Jejunal Atresia.mp.	281
59	Jejuno-Ileal Atresia.mp.	44
60	Malrotation.mp.	2637
61	exp Maxillofacial Abnormalities/ or Maxillofacial Abnormalit*.mp.	29974
62	exp Megacolon/ or Ganglionic Megacolon.mp.	7382
63	exp Mouth Abnormalities/ or Mouth Abnormalit*.mp.	26084
64	exp Neural Tube Defects/ or Neural Tube Defect*.mp.	30773
65	Esophageal Atresia.mp. or exp Esophageal Atresia/ or ?esophageal Atresia.mp.	4500

66	Omphalocele.mp. or exp Hernia, Umbilical/	4485
67	Orofacial Cleft*.mp.	1444
68	Orofacial Cleft*.mp.	1444
69	Pes Cavus.mp.	624
70	Phimosis.mp.	1416
71	Polycystic Kidney Disease.mp.	7924
72	Polydactyly.mp.	3539
73	Preauricular Sinus.mp.	95
74	Rectosigmoid aganglionosis.mp.	19
75	Redundant Neck Fold.mp.	0
76	Renal Anomal*.mp.	1313
77	Spina Bifida.mp.	8781
78	syndactyly.mp	3643
79	Tongue Tie.mp.	269
80	exp Tracheoesophageal Fistula/ or Tracheo-?esophageal fistula*.mp.	3875
81	Umbilical Hernia.mp. or exp Hernia, Umbilical/	4483
82	Undescended Testicle.mp.	268
83	exp Urogenital Abnormalities/ or Genito-urinary Anomalies.mp.	61092
84	Volvulus.mp. or exp Intestinal Volvulus/	9614
85	Webbed Neck.mp.	210
86	Pectus Excavatum.mp.	2126
**87**	**12 or 13 or 14 or 15 or 16 or 17 or 18 or 19 or 20 or 21 or 22 or 23 or 24 or 25 or 26 or 27 or 28 or 29 or 30 or 31 or 32 or 33 or 34 or 35 or 36 or 37 or 38 or 39 or 40 or 41 or 42 or 43 or 44 or 45 or 46 or 47 or 48 or 49 or 50 or 51 or 52 or 53 or 54 or 55 or 56 or 57 or 58 or 59 or 60 or 61 or 62 or 63 or 64 or 65 or 66 or 67 or 68 or 70 or 71 or 72 or 73 or 74 or 75 or 76 or 77 or 78 or 79 or 80 or 81 or 82 or 83 or 84 or 85 or 96**	**674240**
88	LMICs.mp.	3732
89	(low- and middle-income countr*).mp.	17446
90	exp Developing Countries/ or Developing countr*.mp.	126283
91	Low-resource setting*.mp.	4109
92	Underdeveloped Countries.mp.	922
93	low-income countries.mp.	6145
94	middle-income countries.mp.	17641

For peer review only

95	limited resource setting*.mp.	391
96	Africa South of the Sahara.mp. or exp "Africa South of the Sahara"/	209030
97	Sub-Saharan Africa.mp. or exp "Africa South of the Sahara"/	217226
98	Less Resourced communities.mp.	2
99	Afghanistan.mp. or exp AFGHANISTAN/	6919
100	Albania.mp. or exp ALBANIA/	1357
101	Algeria.mp. or exp ALGERIA/	4233
102	American Samoa.mp.	397
103	Angola.mp. or exp ANGOLA/	1623
104	Argentina.mp. or exp ARGENTiNA/	22557
105	Armenia.mp. or exp ARMENIA/	1890
106	Azerbaijan.mp. or exp AZERBAIJAN/	1973
107	Bangladesh.mp. or exp BANGLADESH/	15564
108	Belarus.mp. or exp "Republic of Belarus"/	2704
109	Belize.mp. or exp BELIZE/	967
110	Benin.mp. or exp BENIN/	3906
111	exp BHUTAN/ or Bhutan.mp.	843
112	Bolivia.mp. or exp BOLIVIA/	4085
113	(Bosnia and Herzegovina).mp.	2812
114	Botswana.mp. or exp BOTSWANA/	2698
115	Brazil.mp. or exp BRAZIL/	121099
116	Bulgaria.mp. or exp BULGARIA/	8107

Confidential: For Review Only

117	Burkina Faso.mp. or exp Burkina Faso/	4591
118	Burundi.mp. or exp BURUNDI/	980
119	Cabo Verde.mp. or exp Cabo Verde/	270
120	Cambodia.mp. or exp CAMBODIA/	4833
121	Cameroon.mp. or exp CAMEROON/	7932
122	Central African Republic.mp. or exp Central African Republic/	1216
123	Chad.mp. or exp CHAD/	1317
124	China.mp. or exp CHINA/	269542
125	Colombia.mp. or exp COLOMBIA/	15284
126	Comoros.mp. or exp COMOROS/	506
127	Democratic Republic of the Congo.mp. or exp "Democratic Republic of the Congo"/	5337
128	exp "Democratic Republic of the Congo"/ or DRC.mp.	5579
129	Republic of the Congo.mp. or exp Congo/	7191
130	Costa Rica.mp. or exp Costa Rica/	5719
131	Cote d'Ivoire.mp. or exp Cote d'Ivoire/	3946
132	Ivory Coast.mp. or exp Cote d'Ivoire/	3747
133	Cuba.mp. or exp CUBA/	6860
134	Djibouti.mp. or exp DJIBOUTI/	429
135	Dominica.mp. or exp DOMINICA/	501
136	Dominican Republic.mp. or exp Dominican Republic/	2442
137	Ecuador.mp. or exp ECUADOR/	5777

Confidential: For Review Only

138	exp EGYPT/ or Egypt.mp.	21581
139	El Salvador.mp. or exp El Salvador/	1469
140	Equatorial Guinea.mp. or exp Equatorial Guinea/	481
141	Eritrea.mp. or exp ERITREA/	640
142	Eswatini.mp.	636
143	Ethiopia.mp. or exp ETHIOPIA/	18047
144	Fiji.mp. or exp FIJI/	1950
145	Gabon.mp. or exp GABON/	2127
146	exp GAMBIA/ or Gambia.mp.	3191
147	exp "GEORGIA (REPUBLIC)"/ or exp GEORGIA/ or Georgia.mp.	17363
148	Ghana.mp. or exp GHANA/	11902
149	Grenada.mp. or exp GRENADA/	349
150	Guyana.mp. or exp GUYANA/	1268
151	Haiti.mp. or exp HAITI/	4162
152	Honduras.mp. or exp HONDURAS/	2019
153	India.mp. or exp INDIA/	147631
154	Indonesia.mp. or exp INDONESIA/	16328
155	exp Iran/ or Islamic Republic of Iran.mp.	26832
156	exp IRAQ/ or Iraq.mp.	10229
157	Jamaica.mp. or exp JAMAICA/	4675
158	Jordan.mp. or exp JORDAN/	7350
159	Kazakhstan.mp. or exp KAZAKHSTAN/	3622

Confidential: For Review Only

160	Kenya.mp. or exp KENYA/	21964
161	Kiribati.mp. or exp Micronesia/	2104
162	Democratic People's Republic of Korea.mp. or exp "Democratic People's Republic of Korea"/	300
163	Kosovo.mp. or exp KOSOVO/	962
164	Kyrgyz Republic.mp. or exp Kyrgyzstan/	1312
165	exp Laos/ or Lao PDR.mp.	2206
166	Laos.mp. or exp LAOS/	2903
167	Lebanon.mp. or exp LEBANON/	6114
168	Lesotho.mp. or exp LESOTHO/	762
169	Liberia.mp. or exp LIBERIA/	1920
170	Libya.mp. or exp LIBYA/	1719
171	Madagascar.mp. or exp MADAGASCAR/	5383
172	Malawi.mp. or exp MALAWI/	7731
173	Malaysia.mp. or exp MALAYSIA/	21648
174	Maldives.mp. or exp Indian Ocean Islands/	11983
175	Mali.mp. or exp MALI/	4050
176	Marshall Islands.mp. or exp Micronesia/	2112
177	Mauritania.mp. or exp MAURITANIA/	724
178	Mauritius.mp. or exp MAURITIUS/	1090
179	exp MEXICO/ or Mexico.mp.	60493
180	Micronesia.mp. or exp MICRONESIA/	2424

Confidential: For Review Only

181	Moldova.mp. or exp MOLDOVA/	1021
182	Mongolia.mp. or exp MONGOLIA/	4670
183	Montenegro.mp. or exp MONTENEGRO/	870
184	Morocco.mp. or exp MOROCCO/	7918
185	Mozambique.mp. or exp MOZAMBIQUE/	3923
186	Myanmar.mp. or exp MYANMAR/	4187
187	Namibia.mp. or exp NAMIBIA/	1807
188	Nauru.mp. or exp Micronesia/	2064
189	Nepal.mp. or exp NEPAL/	11583
190	Nicaragua.mp. or exp NICARAGUA/	2202
191	exp NIGER/ or Niger.mp.	13783
192	Nigeria.mp. or exp NIGERIA/	37370
193	North Macedonia.mp.	599
194	Pakistan.mp. or exp PAKISTAN/	24741
195	Papua New Guinea.mp. or exp Papua New Guinea/	5354
196	Paraguay.mp. or exp PARAGUAY/	1676
197	Peru.mp. or exp PERU/	13308
198	Philippines.mp. or exp PHILIPPINES/	12053
199	Romania.mp. or exp ROMANIA/	12624
200	exp Russia/ or Russian Federation.mp.	54195
201	Rwanda.mp. or exp RWANDA/	3505
202	exp AMERICAN SAMOA/ or Samoa.mp. or exp SAMOA/ or exp	1151

Confidential: For Review Only

	"INDEPENDENT STATE OF SAMOA"/	
203	(Sao Tome and Principe).mp.	163
204	Senegal.mp. or exp SENEGAL/	7737
205	Serbia.mp. or exp SERBIA/	5420
206	Sierra Leone.mp. or exp Sierra Leone/	2509
207	Solomon Islands.mp. or exp Melanesia/	6738
208	Somalia.mp. or exp SOMALIA/	2261
209	South Africa.mp. or exp South Africa/	53905
210	South Sudan.mp. or exp South Sudan/	546
211	Sri Lanka.mp. or exp Sri Lanka/	8505
212	Saint Lucia.mp. or exp Saint Lucia/	126
213	(Saint Vincent and the Grenadines).mp.	65
214	exp SUDAN/ or Sudan.mp.	9410
215	Suriname.mp. or exp SURINAME/	1216
216	Swaziland.mp. or exp SWAZILAND/	947
217	Syrian Arab Republic.mp.	85
218	Syria.mp. or exp SYRIA/	3095
219	Tajikistan.mp. or exp TAJIKISTAN/	1034
220	Tanzania.mp. or exp TANZANIA/	15184
221	Thailand.mp. or exp THAILAND/	36416
222	exp Timor-Leste/ or Timor-Leste.mp.	417
223	East Timor.mp.	208

Confidential: For Review Only

224	Togo.mp. or exp TOGO/	1717
225	Tonga.mp. or exp TONGA/	505
226	Tunisia.mp. or exp TUNISIA/	10348
227	exp TURKEY/ or Turkey.mp.	53356
228	Turkmenistan.mp. or exp TURKMENISTAN/	777
229	Tuvalu.mp. or exp Micronesia/	2035
230	Uganda.mp. or exp UGANDA/	16683
231	Ukraine.mp. or exp UKRAINE/	17613
232	Uzbekistan.mp. or exp UZBEKISTAN/	2271
233	Vanuatu.mp. or exp VANUATU/	708
234	Venezuela.mp. or exp VENEZUELA/	7125
235	exp VIETNAM/ or Vietnam.mp.	18647
236	(West Bank and Gaza).mp.	220
237	Republic of Yemen.mp. or exp Yemen/	1396
238	Zambia.mp. or exp ZAMBIA/	6294
239	Zimbabwe.mp. or exp ZIMBABWE/	7682
240	Guatemala.mp. or exp GUATEMALA/	4450
241	Guinea.mp. or exp GUINEA-BISSAU/ or exp EQUATORIAL GUINEA/ or exp GUINEA/ or exp PAPUA NEW/	162475
242	kabul.mp.	452
243	Porto-Novo.mp.	34
244	Hogbonou.mp.	0
245	adjace.mp.	0

Confidential: For Review Only

246	cotonou.mp.	382
247	kutonu.mp.	0
248	ouagadougou.mp.	692
249	ouaga.mp.	4
250	bujumbura.mp	71
251	usumbura.mp.	3
252	phnom penh.mp.	399
253	bangui.mp.	306
254	bangi.mp.	27
255	n'djamena.mp.	111
256	ndjamena.mp.	111
257	fort lamy.mp.	23
258	moroni.mp.	26
259	kinshasa.mp.	1010
260	asmara.mp.	73
261	asmera.mp.	0
262	addis ababa.mp.	1892
263	addis abeba.mp.	82
264	banjul.mp.	84
265	bathurst.mp.	58
266	conakry.mp	297

Confidential: For Review Only

267	bissau.mp.	1267
268	port-au-prince.mp.	320
269	pyongyang.mp.	31
270	monrovia.mp.	430
271	antananarivo.mp.	320
272	tananarive.mp.	83
273	tana.mp.	180
274	lilongwe.mp.	464
275	bamako.mp.	773
276	maputo.mp	543
277	lourenco.mp.	117
278	marques.mp.	476
279	kathmandu.mp.	1612
280	niamey.mp.	289
281	kigali.mp	428
282	freetown.mp	167
283	free-town.mp	6
284	mogadishu.mp	90
285	xamar.mp	0
286	hamar.mp	36
287	muqdisho.mp	0
288	maqadishu.mp	0

Confidential: For Review Only

289	juba.mp	82
290	dodoma.mp	107
291	dar es salaam.mp	1606
292	lome.mo	498
293	kampala.mp	1739
294	harare.mp	1131
295	salisbury.mp	465
296	yerevan.mp	112
297	dhaka.mp	2341
298	dacca.mp	122
299	thimphu.mp	0
300	sucre.mp	1093
301	charcas.mp	2
302	la plata.mp	564
303	chuquisaca.mp	26
304	la paz.mp	761
305	prai.mp	47
306	yaounde.mp	1204
307	jaunde.mp	0
308	brazzaville.mp	615
309	yamoussoukro.mp	21

Confidential: For Review Only

310	cario.mp	2717
311	accra.mp	1393
312	tegucigalpa.mp	81
313	tegus.mp	26
314	new delhi.mp	3169
315	jakarta.mp	1125
316	nairobe.mp	2843
317	south tarawa.mp	14
318	tarawa.mp	36
319	teinainano.mp	0
320	pristina.mp	73
321	prishtina.mp	75
322	bishkek.mp	60
323	pishpek.m	0
324	frunze.mp	95
325	vietiane.mp	302
326	maseru.mp	48
327	nouakchott.mp	96
328	palikir.mp	0
329	chisinau.mp	21
330	kishinev.mp	38
331	rabat.mp	150

Confidential: For Review Only

332	nay pyi taw.mp	8
333	naypyidaw.mp	0
334	nepranytau.mp	0
335	naypyitaw.mp	1
336	kyetpyay.mp	0
337	pyinmana.mp	4
338	kyatpyay.mp	0
339	pyinmana.mp	4
340	yangon.mp	272
341	rangoon.mp	155
342	managua.mp	185
343	abuja.mp	441
344	lagos.mp	2781
345	islamabad.mp	1065
346	port moresby.mp	364
347	moresby.mp	365
348	pom town.mp	0
349	manila.mp	1240
350	apia.mp	74
351	dakar.mp	2196
352	honiara.mp	55

Confidential: For Review Only

353	jayawardenepura.mp	4
354	jayewardenepura.mp	13
355	khartoum.mp	1027
356	mbabane.mp	29
357	embabane.mp	0
358	lobamba.mp	0
359	damascus.mp	286
360	dushanbe.mp	69
361	dyushambe.mp	1
362	stalinabad.mp	2
363	dili.mp	1444
364	kyiv.mp	217
365	kiev.mp	771
366	tashkent.mp	248
367	toshkent.mp	1
368	port vila.mp	19
369	hanoi.mp	1355
370	ha noi.mp	46
371	sana'a.mp	247
372	sanaa.mp	247
373	sana.mp	427
374	lusaka.mp	1091

Confidential: For Review Only

375	ulaanbaatar.mp	247
376	ulan-bator.mp	34
377	luanda.mp	228
378	tbilisi.mp	413
379	amman.mp	702
**380**	**88 or 89 or 90 or 91 or 92 or 93 or 94 or 95 or 96 or 97 or 98 or 99 or 100 or 101 or 102 or 103 or 104 or 105 or 106 or 107 or 108 or 109 or 110 or 111 or 112 or 113 or 114 or 115 or 116 or 117 or 118 or 119 or 120 or 121 or 122 or 123 or 124 or 125 or 126 or 127 or 128 or 129 or 130 or 131 or 132 or 133 or 134 or 135 or 136 or 137 or 138 or 139 or 140 or 141 or 142 or 143 or 144 or 145 or 146 or 147 or 148 or 149 or 150 or 151 or 152 or 153 or 154 or 155 or 156 or 157 or 158 or 159 or 160 or 161 or 162 or 163 or 164 or 165 or 166 or 167 or 168 or 169 or 170 or 171 or 172 or 173 or 174 or 175 or 176 or 177 or 178 or 179 or 180 or 181 or 182 or 183 or 184 or 185 or 186 or 187 or 188 or 189 or 190 or 191 or 192 or 193 or 194 or 195 or 196 or 197 or 198 or 199 or 200 or 201 or 202 or 203 or 204 or 205 or 206 or 207 or 208 or 209 or 210 or 211 or 212 or 213 or 214 or 215 or 216 or 217 or 218 or 219 or 220 or 221 or 222 or 223 or 224 or 225 or 226 or 227 or 228 or 229 or 230 or 231 or 232 or 233 or 234 or 235 or 236 or 237 or 238 or 239 or 240 or 241 or 242 or 243 or 244 or 245 or 246 or 247 or 248 or 249 or 250 or 251 or 252 or 253 or 254 or 255 or 256 or 257 or 258 or 259 or 260 or 261 or 262 or 263 or 264 or 265 or 266 or 267 or 268 or 269 or 270 or 271 or 272 or 273 or 274 or 275 or 276 or 277 or 278 or 279 or 280 or 281 or 282 or 283 or 284 or 285 or 286 or 287 or 288 or 289 or 290 or 291 or 292 or 293 or 294 or 295 or 296 or 297 or 298 or 299 or 300 or 301 or 302 or 305 or 306 or 307 or 308 or 309 or 310 or 311 or 312 or 313 or 314 or 315 or 316 or 317 or 318 or 319 or 320 or 321 or 322 or 323 or 324 or 325 or 326 or 327 or 328 or 329 or 330 or 331 or 332 or 333 or 334 or 335 or 336 or 337 or 338 or 339 or 340 or 341 or 342 or 343 or 344 or 345 or 346 or 347 or 348 or 349 or 350 or 351 or 352 or 353 or 354 or 355 or 356 or 357 or 358 or 359 or 360 or 361 or 362 or 363 or 364 or 365 or 366 or 367 or 368 or 369 or 370 or 371 or 372 or 373 or 374 or 375 or 376 or 377 or 378 or 379**	**1600818**
381	11 AND 87 AND 380	246
